# Supplementary material for: Target choice and exon skipping regulate CRISPR-directed gene editing of NRF2 in head/neck and esophageal cancer cells
Source: Mol Ther Oncol. 2026 Jan 3;34(1):201122. doi: 10.1016/j.omton.2025.201122 (PMC12856552; doi:10.1016/j.omton.2025.201122)
Supplement: Document S1. Figures S1–S6 [file mmc1.pdf]

OMTON, Volume *34*

Supplemental information

Target choice and exon skipping regulate CRISPR-directed gene editing of NRF2 in head/neck and esophageal cancer cells

Natalia Rivera-Torres, Lauren E. Skelly, John A. Rogowskyj, Guadalupe Aguilar, Kelly Banas, Pawel Bialk, and Eric B. Kmiec

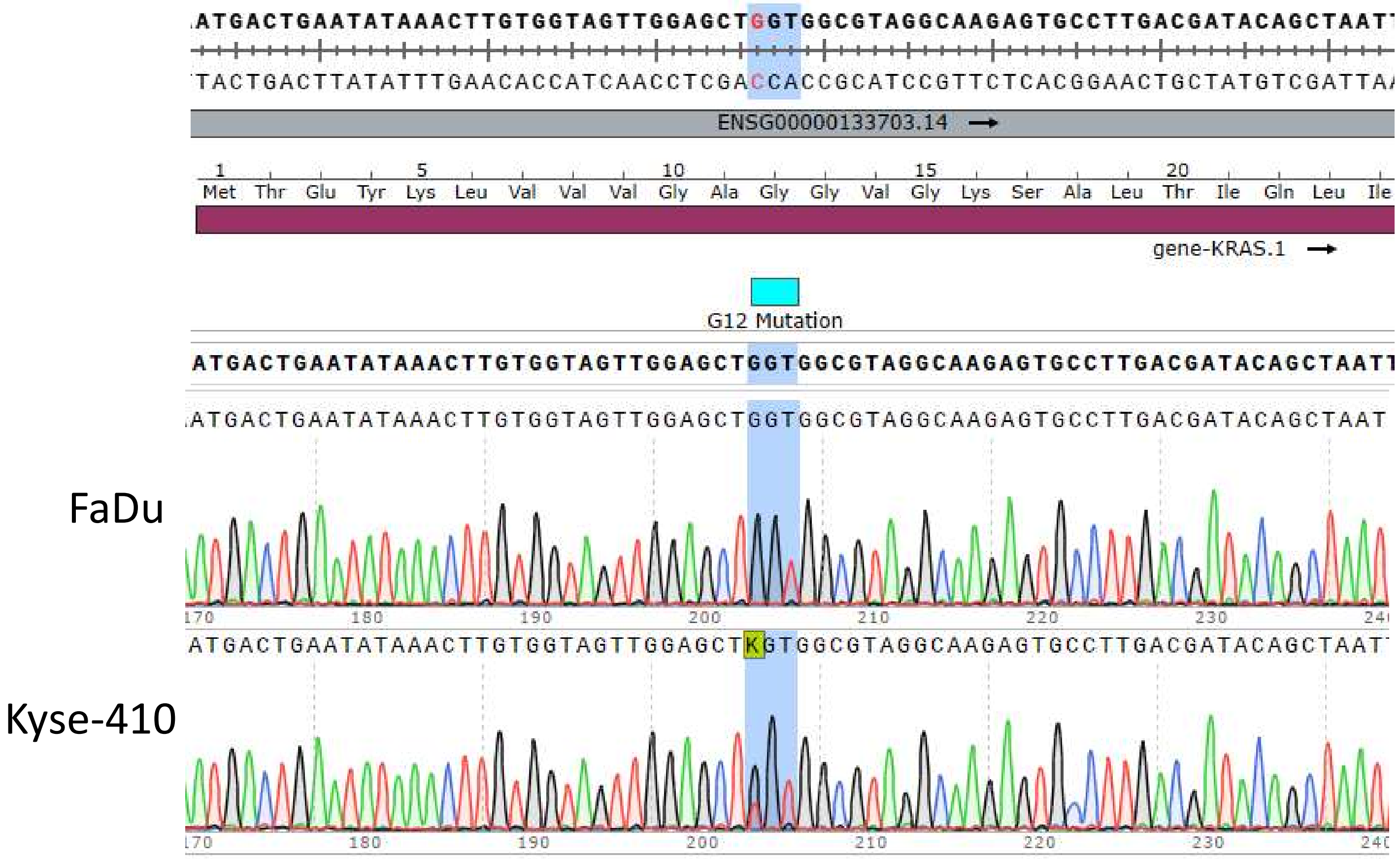

Figure S1. **KRAS Genotyping**. Sanger sequencing results of the KRAS gene in FaDu and KYSE410 cells. The G12D mutation was detected in the KYSE410 cells.

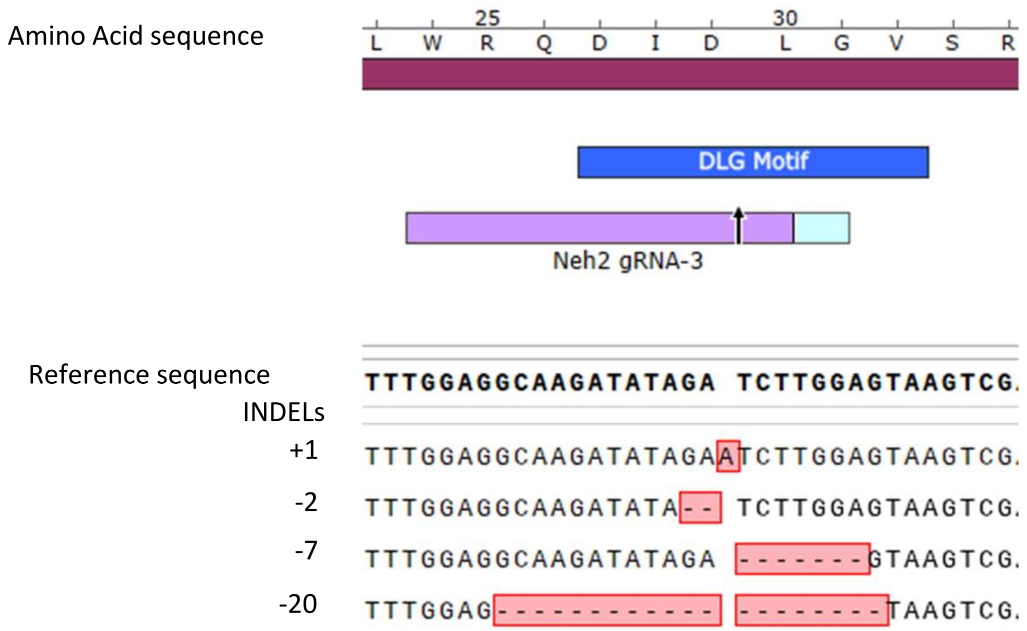

Figure S2. NRF2 interacts with KEAP-1 via the DLG amino acid motif encoded by exon 2. Major INDELS resulting from sgRNA3 targeting directly disrupts sequence.

FaDu

A

sg3

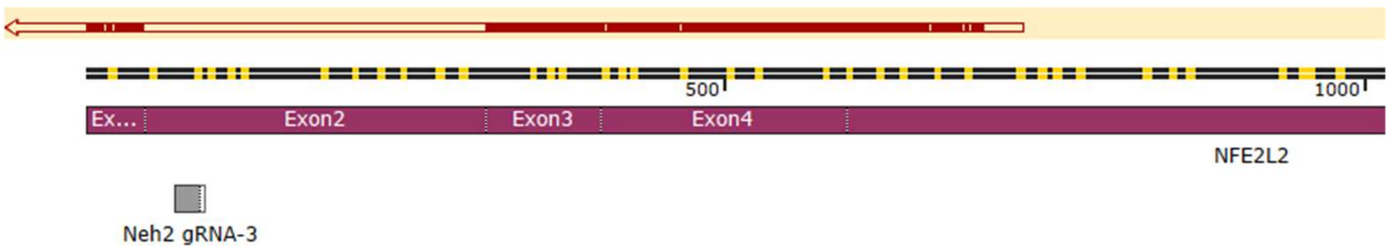

B

sg76

	INDEL —	% ▲	P
G1 -192	-192	38.6	
G1 -9	-9	19.9	
WT	0	19.4	
G1 -1	-1	6.1	
G1 -3	-3	5.9	
G1 -13	-13	5.3	
G1 +1	1	4.8	

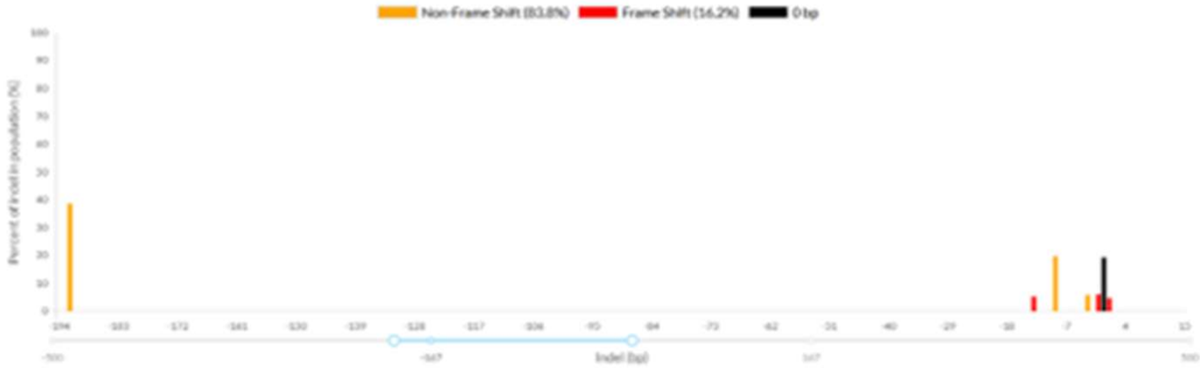

C

sg83

	INDEL —	% ▲
G1 +1	1	43.1
G1 -6	-6	10.7
G1 -4	-4	9.1
WT	0	8.6
G1 -2	-2	7.5
G1 -192	-192	6.3
G1 -1	-1	4.6
G1 -5	-5	3.8
G1 -3	-3	3.1
G1 -13	-13	1.8
G1 -32	-32	1.4

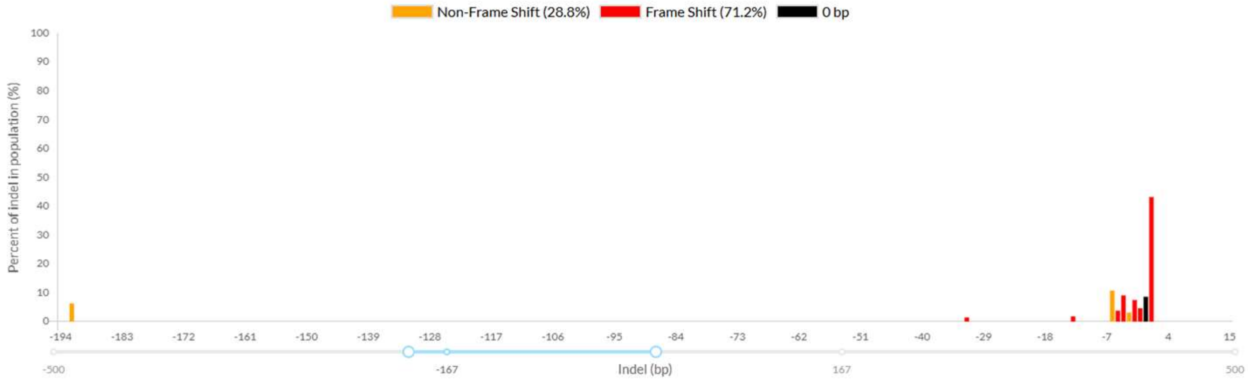

Figure S3. **cDNA sequencing to assess Exon skipping in FADU cells (A)** cDNA from FaDu cells targeted with sg3 in NRF2 was sanger sequenced and aligned to the reference cDNA sequence. cDNA from FaDu cells targeted with sg76 **(B)** or sg83 **(C)** was sanger sequenced and visualized in Decodr (www.decodr.org)

KYSE

A

sg3

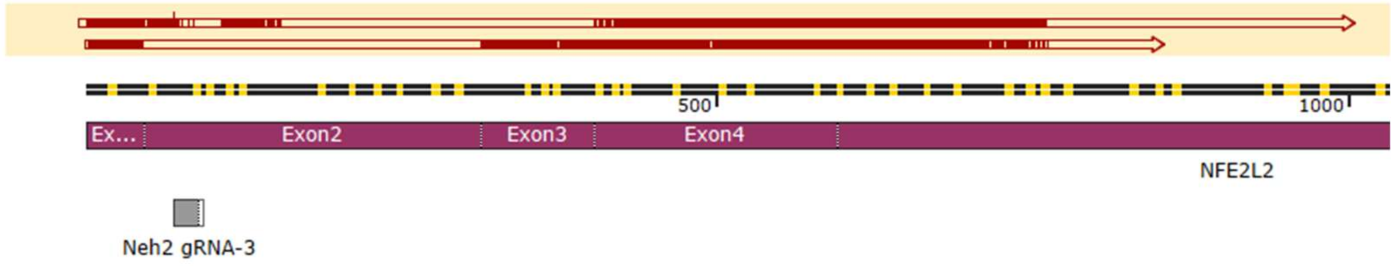

B

sg76

INDEL —		% ▲
G1 -1	-1	41.7
G1 -193	-193	31.2
G1 -10	-10	14.3
G1 -277	-277	7.6
G1 -40	-40	5.1

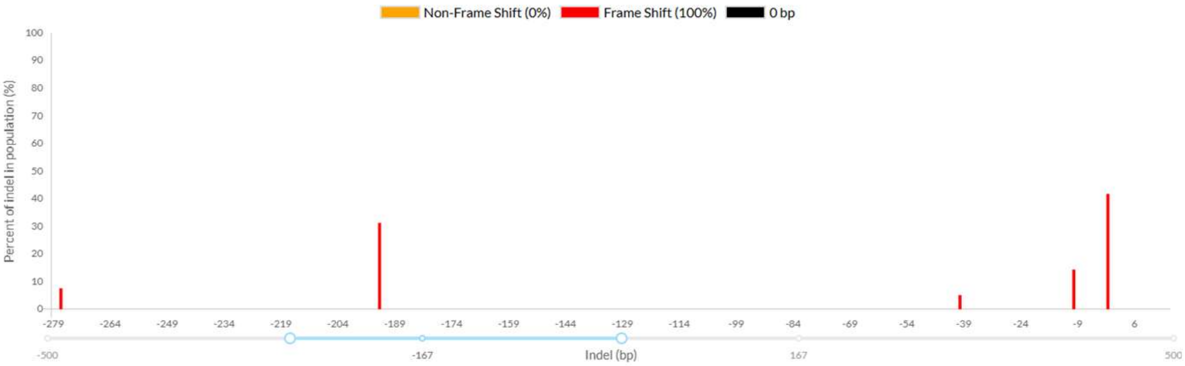

sg83

C

INDEL —		% ▲
G1 +1	1	24.0
G1 -192	-192	19.9
G1 -6	-6	18.6
WT	0	18.4
G1 -4	-4	7.5
G1 -14	-14	6.9
G1 -2	-2	4.7

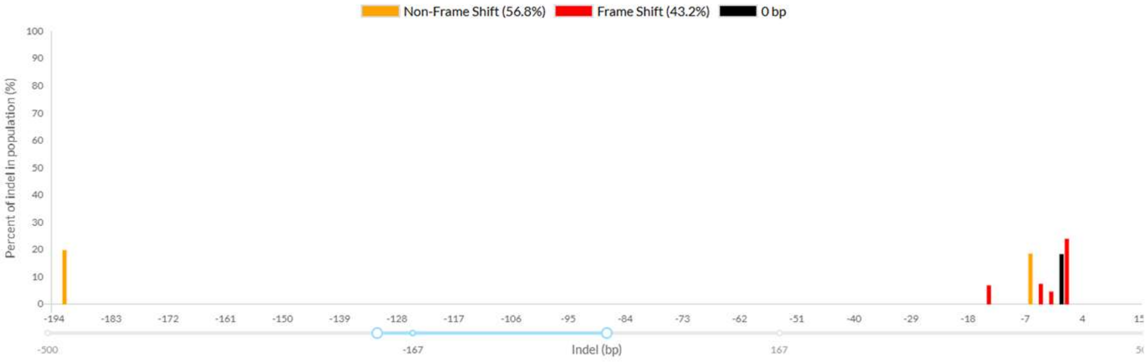

Figure S4.cDNA sequencing to assess Exon skipping in KYSE410 cells.(A) cDNA from KYSE cells targeted with sg3 in NRF2 was sanger sequenced and aligned to the reference cDNA sequence. cDNA from KYSE cells targeted with sg76 **(B)** or sg83 **(C)** was sanger sequenced and visualized in Decodr (www.decodr.org)

Aligned Sequence	INDEL	72hr%	2wk%	%Frameshift 72hr	% Frameshift 2wk
**sg3** TGGAGGCAAGATATAGATCTTGG	Total	85.51	73.45	78.2	74.9
GATTGACATACTTTGGAGGCAAGATATAGATCTTGGAGTAAGTCGAGAAGTATTTGACTT	0	14.49	26.55		
GATTGACATACTTTGGAG--------------------TAAGTCGAGAAGTATTTGACTT	-20	6.07	4.70		
GATTGACATACTTTGGAGGCAAGATATAG**A**ATCTTGGAGTAAGTCGAGAAGTATTTGACT	1	5.70	3.95		
GATTGACATACTTTGGAGGCAAGATATAGA-------GTAAGTCGAGAAGTATTTGACTT	-7	5.30	4.04		
GATTGACATACTTTGGAGGCAAGATATA--TCTTGGAGTAAGTCGAGAAGTATTTGACTT	-2	4.00	4.26		
GATTGACATACTTTGGAGGCAAGATATAGA-CTTGGAGTAAGTCGAGAAGTATTTGACTT	-1	2.87	2.04		
GATTGACATACTTTGGAGGCAAGATAT----CTTGGAGTAAGTCGAGAAGTATTTGACTT	-4	2.67	1.99		
GATTGACATACTTTGGAGGCAAGATATAG-TCTTGGAGTAAGTCGAGAAGTATTTGACTT	-1	2.39	1.72		
GATTGACATACTTTGGAGGCAAG-------------------TCGAGAAGTATTTGACTT	-19	2.25	1.68		
GATTGACATACTTTGGAGGCAAGATATAGA--TTGGAGTAAGTCGAGAAGTATTTGACTT	-2	1.99	1.84		
GATTGACATACTTTGGAGGCAAGATATAGA--------TAAGTCGAGAAGTATTTGACTT	-8	1.84	1.05		
GATTGACATACTTTGGAGGCAAGATATAGA**T**TCTTGGAGTAAGTCGAGAAGTATTTGACT	1	1.77	2.11		
GATTGACATACTTTGGAGGCAAGAT------CTTGGAGTAAGTCGAGAAGTATTTGACTT	-6	1.57	1.29		
GATTGACATACTTTGGAGGCAAGAT-----TCTTGGAGTAAGTCGAGAAGTATTTGACTT	-5	1.37	0.77		
GATTGACATACTTTGGAGGCAAGATAT---TCTTGGAGTAAGTCGAGAAGTATTTGACTT	-3	1.16	1.11		
other		44.57	40.90		
**sg76** GTCACTTGTTCCTGATATTCCCGG	Total	87.94	49.30	68.9	66.6
GGCTACGTTTCAGTCACTTGTTCCTGATAT**T**TCCCGGTCACATCGAGAGC	1	13.10	6.13		
GGCTACGTTTCAGTCACTTGTTCCTGATATTCCCGGTCACATCGAGAGCC	0	12.06	50.70		
GGCTACGTTTCAGTCACTTGTTCCTGATAT-CCCGGTCACATCGAGAGCC	-1	10.97	7.19		
GGCTACGTTTCAGTCACTTGTTCC---------CGGTCACATCGAGAGCC	-9	8.87	5.09		
GGCTACGTTTCAGTCACTTGTTCCTGAT--TCCCGGTCACATCGAGAGCC	-2	3.17	1.81		
GGCTACGTTTCAGTCACTTGT----------CCCGGTCACATCGAGAGCC	-10	2.08	1.06		
GGCTACGTTTCAGTCACTTGTTCCTGAT---------CACATCGAGAGCC	-9	2.00	0.80		
GGCTACGTTTCAGTCACTTGTTCCTGATAT------------CGAGAGCC	-12	1.77	1.21		
GGCTACGTTTCAGTCACTTGTTCC----------GGTCACATCGAGAGCC	-10	1.47	0.34		
GGCTACGTTTCAGTCACTTGTTCCTG---------GTCACATCGAGAGCC	-9	1.39	0.46		
GGCTACGTTTCAGTCACTTGTTCCTGAT---CCCGGTCACATCGAGAGCC	-3	1.39	0.88		
GGCTACGTTTCAGTCACTTGTTCCTG----TCCCGGTCACATCGAGAGCC	-4	1.30	0.74		
GGCTACGTTTCAGTCAC-----------------------ATCGAGAGCC	-23	1.26	0.24		
GGCTACGTTTCAGTCACTTGTTCCTGATAT**AT**TCCCGGTCACATCGAGAG	2	1.09	0.70		
other		38.08	22.66		
**sg83** GTAGCCCCTGTTGATTTAGACGG	Total	86.20	40.95	70.9	67.8
TTCTGTTGCTCAGGTAGCCCCTGTTGATTT**T**AGACGGTATGCAACAGGAC	1	18.19	8.61		
TTCTGTTGCTCAGGTAGCCCCTGTTGATTTAGACGGTATGCAACAGGACA	0	13.80	59.05		
TTCTGTTGCTCAGGTAGCCCCTGTTGA------CGGTATGCAACAGGACA	-6	8.53	4.01		
TTCTGTTGCTCAGGTAGCCCCTGTTGA----GACGGTATGCAACAGGACA	-4	3.81	1.60		
TTCTGTTGCTCAGGTAGCCCCTGTTGATTT**TT**AGACGGTATGCAACAGGA	2	3.20	0.57		
TTCTGTTGCTCAGGTAGCCCCTGTTGATT-AGACGGTATGCAACAGGACA	-1	2.40	1.44		
TTCTGTTGCTCAGGTAGCCCCTGTTGAT--AGACGGTATGCAACAGGACA	-2	2.35	0.83		
TTCTGTTGCTCAGGTAGCCCCTGTTG---TAGACGGTATGCAACAGGACA	-3	1.84	1.00		
TTCTGTTGCTCAGGTAGCCCCTGT-------------ATGCAACAGGACA	-13	1.64	0.37		
TTCTGTTGCTCAGGTAGCCCCTGTTGATTT----------CAACAGGACA	-10	1.43	0.35		
TTCTGTTGCTCAGGTAGCCCCTGTTGA---AGACGGTATGCAACAGGACA	-3	1.25	0.28		
TTCTGTTGCTCAGGTAG---------------ACGGTATGCAACAGGACA	-15	1.21	0.52		
TTCTGTTGCTCAG-------------------ACGGTATGCAACAGGACA	-19	1.19	0.43		
TTCTGTTGCTCAGGTAGCCCCTGTTGATTT-GACGGTATGCAACAGGACA	-1	1.16	0.32		
TTCTGTTGCTCAGGTA----------------------TGCAACAGGACA	-22	1.03	0.69		
other		36.98	19.94		

Figure S5. **Genomic analyses of NRF2 2 weeks post CRISPR/Cas9 targeting.** Genomic DNA from FaDu cells was isolated and amplified across exon 2 and exon 4 of the *NRF2* gene 13 days after transfection. Amplicon was NGS sequenced and analyzed for indels at the CRISPR target site. Raw sequence files were aligned using the software program, CRISPResso2, to display the *NRF2* allele-specific indel pattern

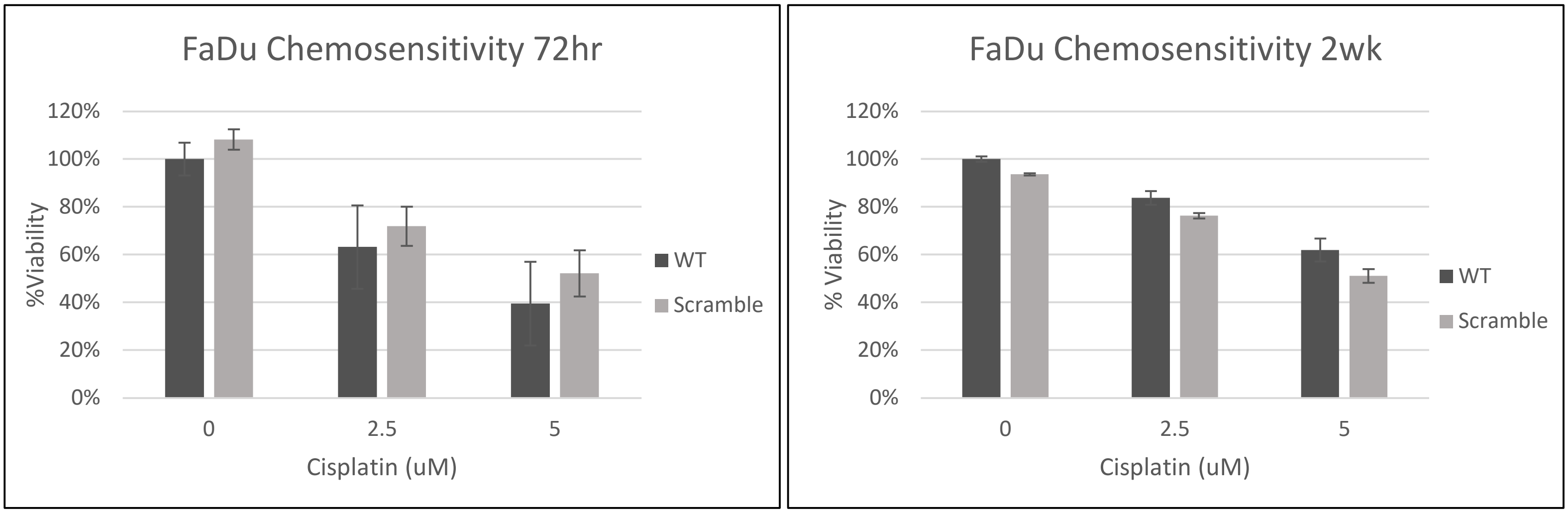

Figure S6. **Sustained Chemosensitivity Testing 2 weeks after CRISPR/Cas9 targeting of NRF2.** Chemosensitivity was measured via CellTiter-Glo® 2.0 Assay. Targeted cells that were maintained in culture for 2 weeks post-transfection was treated with increasing concentrations of cisplatin for 72 hr. and then evaluated for cell viability. The average relative viability of cells normalized to the untreated Wt was graphed. The error bars represent the Coefficient of variance.
