# Supplementary material for: Target choice and exon skipping regulate CRISPR-directed gene editing of NRF2 in head/neck and esophageal cancer cells
Source: Mol Ther Oncol. 2026 Jan 3;34(1):201122. doi: 10.1016/j.omton.2025.201122 (PMC12856552; doi:10.1016/j.omton.2025.201122)
Supplement: Document S2. Article plus supplemental information [file mmc2.pdf]

Molecular Therapy
Oncology
Original Article

Target choice and exon skipping regulate CRISPR-directed gene editing of NRF2 in head/neck and esophageal cancer cells

Natalia Rivera-Torres,[1] Lauren E. Skelly,[1] John A. Rogowskyj,[1] Guadalupe Aguilar,[1] Kelly Banas,[1] Pawel Bialk,[1] and Eric B. Kmiec[1]

[1]ChristianaCare Gene Editing Institute, 550 South College Avenue, Newark, DE 19713, USA

Head and neck cancer (HNC) is the seventh most diagnosed cancer, with a predicted 30% increase annually by 2030. Conventional treatment is often combinatorial involving chemotherapy and radiation therapy, immunotherapy, and surgery, but over time, therapy becomes ineffective as treatment resistance develops. Our laboratory has been advancing a CRISPR-directed gene editing platform as an augmentative therapeutic strategy for squamous cell carcinoma. Our genetic target is *NRF2*, a global transcription regulator involved in response to cellular stress and drug resistance among other cellular functions. In this study, we disable specific endpoints of NRF2 in an attempt to restore chemo-sensitivity in head/neck and esophageal cancer cells. We identify two targeting characteristics that regulate the effectiveness of this approach. The first is the choice of the targeting site reflecting the importance of disabling specific protein domains that achieve specific phenotypic endpoints. The second involves the induction of a molecular rearrangement known as exon skipping that can counterbalance the desired functional outcome even when significant levels of gene disruption are achieved. By considering these experimental parameters, we achieved high levels of gene disruption at the genotypic, phenotypic, and most importantly, functional levels to restore chemosensitivity and significantly reduce tumor cell proliferation.

INTRODUCTION

In 2023, an estimated 66,920 people (49,190 men and 17,730 women) were diagnosed with head and neck cancer (HNC).[1–6] Despite advances in cancer care, chemotherapy and radiation remain prevalent treatment options.[7–10] However, the development of drug resistance in response to these treatments poses a major obstacle to achieving successful outcomes. The molecular mechanism of drug resistance is primarily centered on the upregulation of key genes involved in controlling the efflux of anticancer drugs or directing transcriptional activation of tumor drivers.[8,11–13] Currently, there are few effective means of circumventing drug resistance in solid tumors other than to prescribe another treatment regimen, often with a chance of elevated complex toxicity, or increased dosages of the same therapy. Hence, there is a pressing need to develop tools to address drug resistance and enable a more widespread use of standard of care for better disease prognosis.

We are developing CRISPR-Cas gene editing-based therapeutic platforms that disable genes involved in drug resistance in solid tumors. This strategy addresses the core of the treatment resistance problem at the genetic level. Gene editing takes place when the Cas9 nuclease is directed by a single-guide RNA (sgRNA) to modify a specific chromosomal DNA sequence by inducing a sequence-specific double-strand break (DSB). DSBs are predominantly resolved via the error-prone non-homologous end joining repair mechanism, which can induce insertions or deletions (indels) resulting in genetic disruption.

Our genetic target is the *NRF2* (NFE2L2) gene, a master transcription regulator responsible for resistance to cancer therapy in squamous cell carcinomas of the head, neck, esophagus, and lungs.[14–18] Under normal conditions, NRF2 is sequestered in the cytosol by its binding partner, KEAP-1. KEAP-1 regulates NRF2 by recruiting components of the (E3) ubiquitin ligase pathway. When oxidative stress is introduced, NRF2 translocates to the nucleus and activates downstream targets involved in many aspects of oncogenic progression.[19] While transient activation in response to stress is beneficial for survival in normal cells, constitutive NRF2 activation in cancer cells has deleterious effects on the host by amplifying the antioxidant and detoxification capability of cancer cells, driving metabolic reprogramming to establish cellular metabolic processes that accelerate cell proliferation.[20,21] This, in turn, confers therapeutic resistance and activates an aggressive tumorigenic phenotype.[22] Mechanisms of NRF2 activation include somatic mutation, copy number variation (gain of function or amplification), and changes in the interactions with KEAP1 (loss of function or deletion). Mutations disrupt KEAP1 binding and lead to constitutive expression of NRF2 in

Received 18 March 2025; accepted 31 December 2025;
https://doi.org/10.1016/j.omton.2025.201122.

Correspondence: Eric B. Kmiec, ChristianaCare Gene Editing Institute, 550 South College Avenue, Newark, DE 19713, USA.
E-mail: eric.b.kmiec@christianacare.org

© 2026 The Author(s).
Published by Elsevier Inc. on behalf of The American Society of Gene and Cell Therapy.
This is an open access article under the CC BY license (http://creativecommons.org/licenses/by/4.0/).

A

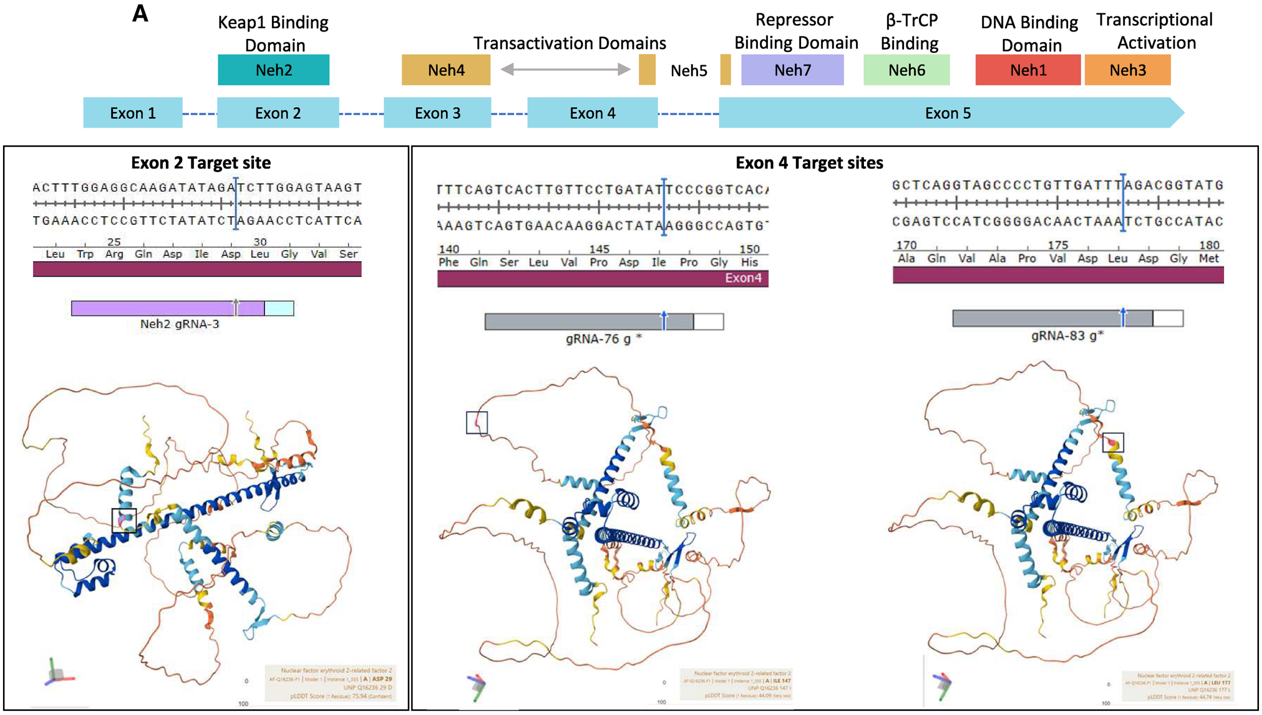

B

Guide RNA	Sequence	GRCh38 Position	0 MM	1 MM	2 MM	3 MM	4MM
sg3	TGGAGGCAAGATATAGATCT TGG	chr2: 177234224	1 (onT)	0	1	33	425
sg76	GTCACTTGTTCCTGATATTCC CGG	chr2: 177232539	1 (onT)	1	1	10	136
sg83	GTAGCCCCTGTTGATTTAGA CGG	chr2: 177232449	1 (onT)	1	1	16	189

C

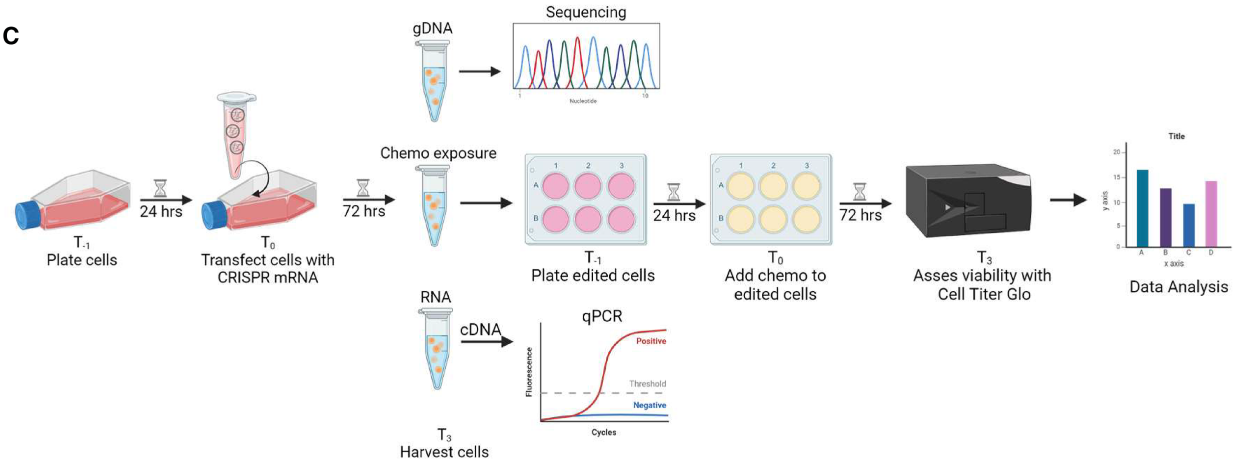

(legend on next page)

cancer cells.[20,23] These mutations are frequently found in solid tumors especially in the head and neck (25%), lung (11%), colon (8%), liver (9%), and breast (2%). NRF2 target genes include antioxidant genes and phase II enzymes such as heme oxygenase-1 (*HMOX-1*), NAD(P)H: quinone oxidoreductase 1 (*NQO1*), glutathione S-transferase, and glutathione peroxidase.[24,25] Hence, NRF2 qualifies as an attractive molecular target for several reasons: (1) it is a far upstream component of the pathway that leads to resistance to many forms of cancer therapy, (2) knocking out a master regulator can have a potential downstream effect on multiple genes that block drug action, and (3) it is heavily upregulated during tumorigenesis.

We demonstrated previously that gene editing can be utilized as a biomolecular tool to disable specific genes involved in drug resistance of lung tumors.[26,27] Further studies conducted in xenograft mouse models demonstrate a significant reduction in tumor cell growth and better survival in animals treated with a combination of CRISPR-Cas and low-dose chemotherapy.[26,28] These results prompted an expansion of proof-of-concept experiments to other squamous cell carcinomas such as HNC and esophageal cancer. Our work suggests that such a genetic strategy can be coupled with standard chemotherapy (and others) reducing side effects from primary treatment, improving quality of life, and prolonging survival.

RESULTS

The experimental system and target sites

The *NRF2* gene is segmented into five exons, each encoding important functional protein domains (Figure 1A). Exon 2 is well known because it is the domain (Neh2) that enables interaction with KEAP1.[29–31] Exon 4 links the Neh4 and Neh5 domains, which are critically important for the overall function of NRF2 in its role as a master regulator and transcription factor. We have previously demonstrated that both exons can be targeted by CRISPR-Cas, resulting in the de-activation of NRF2 gene function in adenocarcinoma and squamous cell carcinoma of the lung.[26,27,32] Exon 2 harbors a unique mutation, R34G, that is found in a subset of squamous cell patients, and this mutation creates a unique PAM site enabling tumor-specific CRISPR activity.[27,33,34] Exon 4 has also been exploited as a target site with an initial disruption site in A549 lung cancer cells severely reducing NRF2 activity and further validated in FaDu cells.[26,35]

The objective of this study was to evaluate the functional outcomes of CRISPR-directed gene editing through the disruption of NRF2 and the restoration of sensitivity to chemotherapy in bulk populations. Previous results guided our decision to design sgRNAs that target either exon 2 or 4, for disruption of NRF2 in HNC cells. Unlike the case of squamous cell carcinoma of the lung, the R34G mutation exists in HNC cells at a much lower frequency than in the lung.[33] So, here we targeted a region of the normal NRF2 sequence, sg3 (TGGAGGCAAGATATAGATCT) at the DLG motif designed to disrupt exon 2. In parallel, two guide (gRNAs), sg76 (GTCACTTGTTCCTGATATTCC) and sg83 (GTAGCCCCTGTT GATTTAGA), were designed to target exon 4. sg3 and sg83 target the alpha loop structures of the NRF2 protein, while sg76 targets a disorganized region. We also performed *in silico* off-target assessments using Cas-OFFinder for all three gRNAs (Figure 1B).[36]

Figure 1C outlines the work plan carried out on FaDu cells, established from a punch biopsy of a hypopharyngeal tumor removed from a 56-year-old, white male patient with squamous cell carcinoma.[37] First, we confirmed that the DNA sequence of exon 2 and exon 4 of the NRF2 gene in these cells was not mutated and amenable to targeting with CRISPRs sg3, sg76, or sg83, respectively (data not shown). While this may seem like a trivial matter, it is not, particularly with mutator phenotypes in transformed cells. Cells were plated 24 h prior to the introduction of the CRISPR-Cas complex, with the respective sgRNA, and delivered into the cells by the Lipofectamine MessengerMAX mRNA Transfection Reagent. Seventy-two hours after exposure to CRISPR-Cas, a sample of the cells was harvested for genomic sequencing and RNA isolation, while the remaining population was replated and recovered for 24 h. After that time, the cell population was treated with cisplatin or 5-fluorouracil (5-FU) at various doses. Cisplatin and 5-FU were selected based on NCCN (National Comprehensive Cancer Network) treatment guidelines as cisplatin is the standard chemotherapy drug, clinically recommended as part of chemoradiation alone or in combination with 5-FU.[3,38,39] Three days after treatment, the cells were harvested and the cell viability measured. This conservative workflow was established to assess how gene editing takes place prior to the introduction of the chemotherapeutic agent driving the restoration of sensitivity.

This experimental plan allows for more direct analyses of the impact of CRISPR activity, which we recognize could continue in the presence of chemotherapy. We decided to use a single dose of the drug at a single point in time, a conservative approach to drug testing in cell culture, although multiple rounds of chemotherapy at lower therapeutic doses could be more effective in a clinical setting.

For all gene editing studies from bench to bedside, it is important to employ a functional assay, accompanied by an internal functional control, to assess the efficiency of CRISPR-Cas activity. In this case, NRF2 disruption can be measured functionally through qPCR analysis of some of its well-established and characterized target genes *NQO1*, *HMOX1*, and *GCLC*. These genes have been used as a

Figure 1. NRF2 target sites and experimental systems

(A) Structural domains of the NRF2 protein aligned to the exons of the NRF2 gene. Three guide RNAs were designed to cleave the NRF2 gene. Sg3 targets within the DLG motif in exon 2, while sg76 and sg83 target within the transactivation domain in exon 4. String protein models created in AlphaFold (https://alphafold.ebi.ac.uk). (B) *In silico* off-target analysis was performed using Cas-OFFinder (Bae et al., 2014) for sg3, sg76, and sg83, allowing up to four mismatches and recognizing both NAG and NGG PAMs (GRCh38: Genome Reference Consortium human build 38; MM: mismatch; OnT: On-target). (C) Experimental CRISPR-Cas9 delivery workflow for targeting NRF2.

A

Aligned Sequence	INDEL	%	Total INDEL %	Frameshift %
**FADU** sg3 TGGAGGCAAGATATAGATCTTGG				
GATTGACATACTTTGGAGGCAAGATATAGATCTTGGAGTAAGTCGAGAAGTATTTGACTT	0	23.64	76.4	76.34
GATTGACATACTTTGGAG--------------------TAAGTCGAGAAGTATTTGACTT	-20	6.70		
GATTGACATACTTTGGAGGCAAGATATAGA-------GTAAGTCGAGAAGTATTTGACTT	-7	4.93		
GATTGACATACTTTGGAGGCAAGATATAG**A**ATCTTGGAGTAAGTCGAGAAGTATTTGACT	1	4.46		
GATTGACATACTTTGGAGGCAAGATATA--TCTTGGAGTAAGTCGAGAAGTATTTGACTT	-2	3.71		
GATTGACATACTTTGGAGGCAAGATAT----CTTGGAGTAAGTCGAGAAGTATTTGACTT	-4	2.50		
GATTGACATACTTTGGAGGCAAGATATAGA-CTTGGAGTAAGTCGAGAAGTATTTGACTT	-1	2.14		
GATTGACATACTTTGGAGGCAAGATATAGA--TTGGAGTAAGTCGAGAAGTATTTGACTT	-2	2.09		
GATTGACATACTTTGGAGGCAAG-------------------TCGAGAAGTATTTGACTT	-19	1.89		
GATTGACATACTTTGGAGGCAAGATATAG-TCTTGGAGTAAGTCGAGAAGTATTTGACTT	-1	1.70		
GATTGACATACTTTGGAGGCAAGATATAGAT**T**CTTGGAGTAAGTCGAGAAGTATTTGACT	1	1.49		
GATTGACATACTTTGGAGGCAAGAT------CTTGGAGTAAGTCGAGAAGTATTTGACTT	-6	1.32		
GATTGACATACTTTGGAGGCAAGATATAGA--------TAAGTCGAGAAGTATTTGACTT	-8	1.02		
GATTGACATACTTTGGAGGCAAGATATAG---------TAAGTCGAGAAGTATTTGACTT	-9	1.00		
others		65.05		

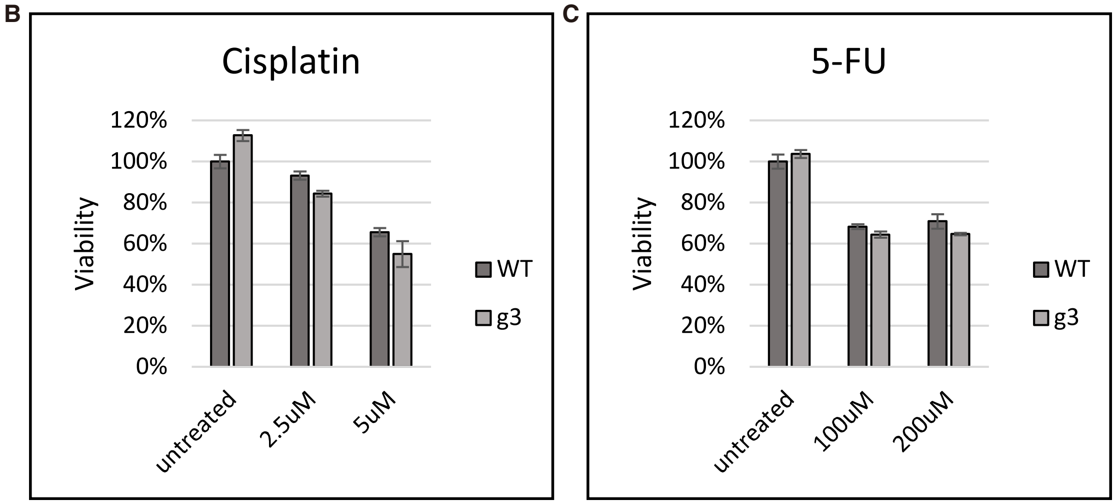

Figure 2. Genetic disruption of NRF2 at exon 2 in FaDu cells

(A) Genomic analyses of NRF2 after CRISPR targeting. Genomic DNA from FaDu cells was isolated and amplified across exon 2 of the *NRF2* gene. Amplicon was sequenced by next-generation sequencing (NGS) and analyzed for indels at the CRISPR target site. Raw FASTQ sequence files were aligned using the software program, CRISPResso2, to display the *NRF2* allele-specific indel pattern and frameshift percent of the targeted outcomes. (B and C) Chemosensitivity testing in response to NRF2 exon 2 targeting. Chemosensitivity was measured via CellTiter-Glo 2.0 Assay. Targeted cells were treated with increasing concentrations of cisplatin or 5-FU for 72 h and then evaluated for cell viability. The average relative viability of cells normalized to the untreated WT was graphed. The error bars represent the coefficient of variance.

standard measure of NRF2 activity in response to oxidative damage and drug resistance in cancer cells.[22,40] Activation of these genes by NRF2 increases the cytoprotective roles against oxidative stress and can promote cancer progression by driving tumor cell proliferation and metastasis.[41]

Genetic disruption of NRF2 at exon 2 in FaDu cells

Figure 2A displays the indel profile generated by the sg3/Cas9 complex after 72 h of incubation. The total indel population exceeded 76%, and more importantly, within that profile, over 76% (of the 76% total) were frameshift mutations. Under any circumstances, inducing allelic knockout of 76% in FaDu cells reflects high levels of gene editing. However, despite high levels of genetic disruption, targeted cells were rendered minimally chemo-sensitive (Figures 2B and 2C) and retained the same level of drug resistance as untreated cells. These results were surprising considering genetic disruption at the NRF2 R34G gene mutation in exon 2 resulted in significant restoration of chemosensitivity in squamous cell carcinoma of the lung.[27,34]

Genetic disruption of NRF2 at exon 4 in FaDu cells

Following the unexpected results of NRF2 exon 2 disruption with sg3, we targeted exon 4 with two different specific gRNAs, sg76 and sg83. Once again, gene editing was assessed after 72 h, and the indel profiles are presented in Figure 3A. The degree of NRF2 gene disruption paralleled levels seen with sg3, and the percentage of

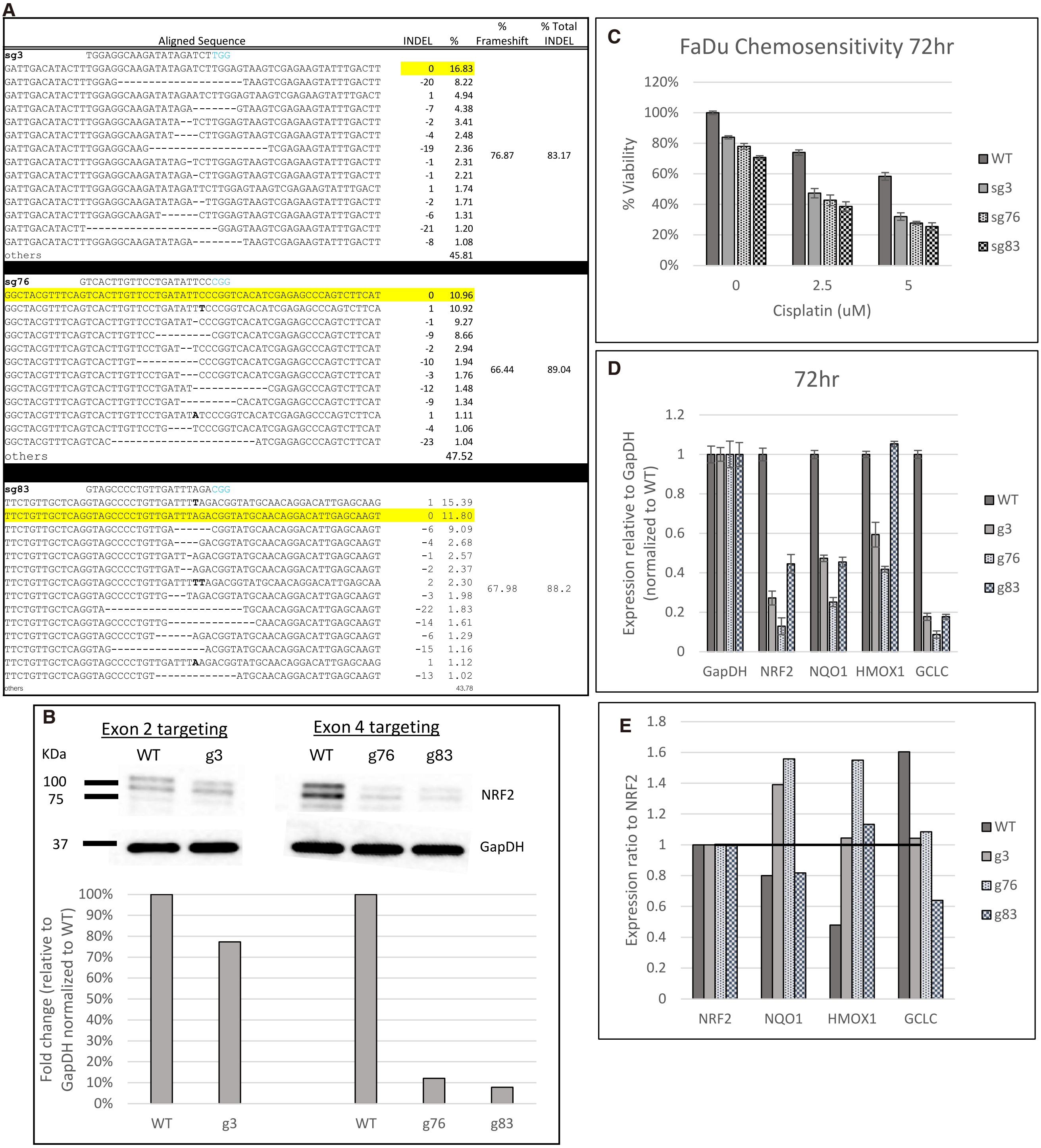

Figure 3. Genetic disruption of *NRF2* at exon 4 in FaDu cells

(A) Genomic analyses of after CRISPR targeting. Genomic DNA from FaDu cells was isolated and amplified across exon 4 of the *NRF2* gene. Amplicon was NGS sequenced and analyzed for indels at the CRISPR target site. Raw sequence files were aligned using the software program, CRISPResso2, to display the *NRF2* allele-specific indel pattern and frameshift percent of the targeted outcomes. (B) Protein analysis by western blot. Protein was isolated from edited cells, and NRF2 expression was analyzed relative to GapDH and normalized to the representative wild-type control from each transfection. (C) Chemosensitivity testing in response to *NRF2* exon 4 targeting. Chemosensitivity was measured via CellTiter-Glo 2.0 Assay. Targeted cells were treated with increasing concentrations of cisplatin for 72 h and then evaluated for cell viability.

(legend continued on next page)

frameshift (Indel Code) was also similar. NRF2 is also knocked down at the protein level at the 72-h time point (Figure 3B). The antibody used (see details in material and methods) binds to lysine 550, encoded by exon 5 near the 3′ UTR/C terminus.[42] Therefore, the epitope is representative of total NRF2 protein independent of the three target sites and their outcomes. Compared to a wild-type (WT) control, exon 2 sg3 shows 30% knockdown, while 90% knockdown was achieved by targeting exon 4 with sg76 and sg83. We then added cisplatin for 72 h and measured the viability of the targeted and the untargeted cells. Figure 3C displays the results. Untargeted cells, or those without the addition of cisplatin, were processed the same as targeted cells treated with the drug. FaDu cells edited by sg3, sg76, or sg83, followed by a single dose of cisplatin, exhibited a reduction in viability and restoration of chemosensitivity, with exon 4-targeted cells gaining higher sensitivity than exon 2. We also observed that exon 4-edited cells showed reduced viability from just the disruption of NRF2 alone compared to the exon 2-targeted cells. These data suggest that the choice of the target site within NRF2 elicits significant functional disruption. In addition, *NQO1* and *GCLC* expression levels were diminished when FaDu cells were transfected with sg83 (Figure 3D). To better understand NRF2 function in each knockdown condition, we divided the relative expression from Figure 3D by the NRF2 knockdown level in each gRNA condition to produce a ratio of NRF2 expression to each downstream target (Figure 3E). Similarly to the protein assessment, the primers used for *NRF2* qPCR bind to exon 5, which is presumably unaffected. While *NRF2* levels are largely reduced in all three targeted conditions, g3 and g76 do not show a reduction in expression of downstream target genes relative to *NRF2* levels. This analysis provides further evidence to support that the impact of genetic disruption of *NRF2* alone is sufficient to disrupt its transcription activation function.

Genetic disruption of NRF2 at exon 2 or exon 4 in KYSE-410 cells

Next, we wanted to test the applicability of this platform approach in other cancer types. So, we executed the same protocol on KYSE-410 cells, which originate from a poorly differentiated invasive esophageal squamous cell carcinoma resected from the cervical esophagus of a 51-year-old Japanese man prior to treatment.[43] As done in the FaDu cells, we confirmed that the DNA sequence of exons 2 and 4 were not mutated and amenable to targeting with CRISPRs sg3, sg76, or sg83, respectively (data not shown). Plated cells were allowed to recover for 24 h prior to transfection with the same CRISPR complexes as described in Figure 3. Seventy-two hours post-transfection, the indel profiles revealed a high degree of gene editing activity approaching 100% (97%) with all three gRNAs, sg3, sg76, and sg83 (Figure 4A). These overall levels are generally considered to be a complete genetic disruption of a mammalian gene. The percentage of frameshift mutations created by the action of these gRNAs mimics those levels seen previously in FaDu cells. The response of targeted cells to cisplatin reflected the levels of chemosensitivity restored by sg76 and sg83 at 15 and 30 μM of cisplatin, but notably, sg3 only showed sensitivity at 30 μM (Figure 4B). As previously reported, cisplatin concentrations were adjusted to account for the relative resistance in KYSE-410 cells.[44] This cell line is known to have a KRAS G12C mutation (Figure S1) that could contribute to sustained chemoresistance by activating alternative pathways or altering NFR2 activity by modulating its transcriptional activity.[45,46] The expression levels of *NRF2, NQO1, HMOX1*, and *GCLC* were also assessed, as shown in Figure 4C. Exon 4 targeting by sg83 results in lower levels of *NQO1* and *GCLC* expression than the exon 2 (sg3)- or exon 4 (sg76)-targeted cells. To better characterize these results, the ratio of *NRF2* expression to each downstream target was calculated, as shown in Figure 4D. This analysis further supports that disruption of *NRF2* in exon 4 has a higher impact on overcoming chemosensitivity by disrupting its function of activating downstream genes.

These results collectively emphasize the importance of choosing properly the targeting site in the gene and the domain of the encoded protein to achieve the desired phenotypic and functional outcome. In addition, the internal consistency among the editing activity of the specific gRNAs, where disruption was achieved above 85% in both FaDu and KYSE 410 cells, codifies the identification of sg76 and sg83 as useful biomolecules for the disruption of NRF2.

Exon skipping can regulate functional outcomes of NRF2 gene disruption

Genomic modification at unintended sites is at the heart of the safety concerns surrounding CRISPR-directed gene editing. While much emphasis is placed on off-site mutagenesis, more often, on-site alterations such as genomic translocations, inversions, gene conversion, and unexpected disruption of mRNA sequence,[39,43] are likely more impactful. The difference in chemosensitivity between targeting exon 2 and exon 4 is intriguing, so we wanted to explore if a molecular rearrangement, exon skipping, which we had previously identified within the *NRF2* gene, was in part responsible for this difference.[32,47]

KEAP-1 regulates NRF2 in the cells by binding to DLG and ETGE amino acid motifs encoded within exon 2. The sg3 cut site directly disrupts the DLG motif by producing indels, inhibiting the binding of KEAP-1, and leading to an increased intranuclear concentration of NRF2 that can now act on its downstream targets, thereby sustaining chemoresistance (Figure S2). The same outcomes can be produced when the NRF2 undergoes exon skipping of exon 2. In this case, both binding sites for KEAP-1 are lost. Since we know NRF2

The average relative viability of cells normalized to the untreated WT was graphed. The error bars represent the coefficient of variance. (D) qPCR gene expression analysis. RNA was isolated from targeted cells and converted to cDNA. Relative gene expression of *NRF2*, *NQO1*, *HMOX1*, and *GCLC* was measured through qPCR and analyzed using the Pfaffl method. Relative gene expression normalized to the WT cells was graphed. The error bars represent the coefficient of variance. (E) Ratio of NRF2 expression to downstream targets. Values from (C) were divided by NRF2 expression values for each condition to show the ratio of *NRF2* to *NQO1*, *HMOX1*, and *GCLC* when *NRF2* expression is reduced.

A

Aligned Sequence	INDEL	% INDEL	% Total INDEL	% Frameshift
**sg3** TGGAGGCAAGATATAGATCTTGG				
GATTGACATACTTTGGAG--------------------TAAGTCGAGAAGTATTTGACTT	-20	18.64		
GATTGACATACTTTGGAGGCAAG-------------------TCGAGAAGTATTTGACTT	-19	6.05		
GATTGACATACTTTGGAGGCAAGATATAGA-------GTAAGTCGAGAAGTATTTGACTT	-7	5.51		
GATTGACATACTTTGGAGGCAAGATATA--TCTTGGAGTAAGTCGAGAAGTATTTGACTT	-2	3.80		
GATTGACATACTTTGGAGGCAAGATATAGA**A**TCTTGGAGTAAGTCGAGAAGTATTTGACT	1	3.33		
GATTGACATACTT---------------------GGAGTAAGTCGAGAAGTATTTGACTT	-21	2.64		
GATTGACATACTTTGGAGGCAAGATAT----CTTGGAGTAAGTCGAGAAGTATTTGACTT	-4	2.45		
GATTGACATACTTTGGAGGCAAGATATAGATCTTGGAGTAAGTCGAGAAGTATTTGACTT	0	2.27	97.73	77.06
GATTGACATACTTTGGAGGCAAGATATAGA--TTGGAGTAAGTCGAGAAGTATTTGACTT	-2	2.03		
GATTGACATACTTTGGAGGCAAGATATAG---------TAAGTCGAGAAGTATTTGACTT	-9	1.70		
GATTGACATACTTTGGAGGCAAGATATAGA-CTTGGAGTAAGTCGAGAAGTATTTGACTT	-1	1.26		
GATTGACATACTTTGGAGGCAAGATATAG-TCTTGGAGTAAGTCGAGAAGTATTTGACTT	-1	1.22		
GATTGACATACTTTGGAGGCAAGATATAGA**T**TCTTGGAGTAAGTCGAGAAGTATTTGACT	1	1.18		
GATTGACATACTTTGGAGGCAAGAT------CTTGGAGTAAGTCGAGAAGTATTTGACTT	-6	1.10		
GATTGACATACTTTGGAGGCAAG---------------TAAGTCGAGAAGTATTTGACTT	-15	1.00		
Others		45.8		
**sg76** GTCACTTGTTCCTGATATTCCCGG				
GGCTACGTTTCAGTCACTTGTTCC---------CGGTCACATCGAGAGCCCAGTCTTCAT	-9	16.96		
GGCTACGTTTCAGTCACTTGTTCCTGATAT**T**TCCCGGTCACATCGAGAGCCCAGTCTTCA	1	11.24		
GGCTACGTTTCAGTCACTTGTTCCTGATAT-CCCGGTCACATCGAGAGCCCAGTCTTCAT	-1	3.86		
GGCTACGTTTCAGTCACTTGTTCCTGAT---------CACATCGAGAGCCCAGTCTTCAT	-9	2.78		
GGCTACGTTTCAGTCACTTGTTCCTGATATTCCCGGTCACATCGAGAGCCCAGTCTTCAT	0	2.33		
GGCTACGTTTCAGTCACTTGTTCCTG---------GTCACATCGAGAGCCCAGTCTTCAT	-9	2.25		
GGCTACGTTTCAGTCACTTGTTCCTGAT--TCCCGGTCACATCGAGAGCCCAGTCTTCAT	-2	2.14	97.67	54.40
GGCTACGTTTCAGTCACTTGTTCC----------GGTCACATCGAGAGCCCAGTCTTCAT	-10	2.08		
GGCTACGTTTCAGTCAC-----------------------ATCGAGAGCCCAGTCTTCAT	-23	1.97		
GGCTACGTTTCAGTCACTTGT----------CCCGGTCACATCGAGAGCCCAGTCTTCAT	-10	1.95		
GGCTACGTTTCAGTCACTTGTTCCTG----------TCACATCGAGAGCCCAGTCTTCAT	-10	1.36		
GGCTACGTTTCAGTCACTTGTTCCTGATA**TA**TTCCCGGTCACATCGAGAGCCCAGTCTTC	2	1.34		
GGCTACGTTTCAGTCACTTGTTCCTG----TCCCGGTCACATCGAGAGCCCAGTCTTCAT	-4	1.14		
GGCTACGTTTCAGTCACTTGTTCCTGA---------------------------------	-150	3.01		
others		48.61		
**sg83** GTAGCCCCTGTTGATTTAGACGG				
TTCTGTTGCTCAGGTAGCCCCTGTTGA------CGGTATGCAACAGGACATTGAGCAAGT	-6	17.95		
TTCTGTTGCTCAGGTAGCCCCTGTTGATT**T**TAGACGGTATGCAACAGGACATTGAGCAAG	1	13.46		
TTCTGTTGCTCAGGTAGCCCCTGTTG--------------CAACAGGACATTGAGCAAGT	-14	3.70		
TTCTGTTGCTCAGGTAGCCCCTGTTGATT**TT**TAGACGGTATGCAACAGGACATTGAGCAA	2	3.20		
TTCTGTTGCTCAGGTAGCCCCTGTTGATTTAGACGGTATGCAACAGGACATTGAGCAAGT	0	2.85		
TTCTGTTGCTCAGGTAGCCCCTGT-------------ATGCAACAGGACATTGAGCAAGT	-13	2.29		
TTCTGTTGCTCAGGTAGCCCCTGTTGATT-AGACGGTATGCAACAGGACATTGAGCAAGT	-1	1.88	97.15	60.8
TTCTGTTGCTCAGGTAGCCCCTGTTGA----GACGGTATGCAACAGGACATTGAGCAAGT	-4	1.77		
TTCTGTTGCTCAGGTA----------------------TGCAACAGGACATTGAGCAAGT	-22	1.75		
TTCTGTTGCTCAGGTAGCCCCTGTTGAT--AGACGGTATGCAACAGGACATTGAGCAAGT	-2	1.59		
TTCTGTTGCTCAGGTAGCCCCTGTTG---TAGACGGTATGCAACAGGACATTGAGCAAGT	-3	1.54		
TTCTGTTGCTCAGGTAGCCCCTG---------ACGGTATGCAACAGGACATTGAGCAAGT	-9	1.28		
TTCTGTTGCTCAGGTAGC-----------------------AACAGGACATTGAGCAAGT	-23	1.05		
others		45.69		

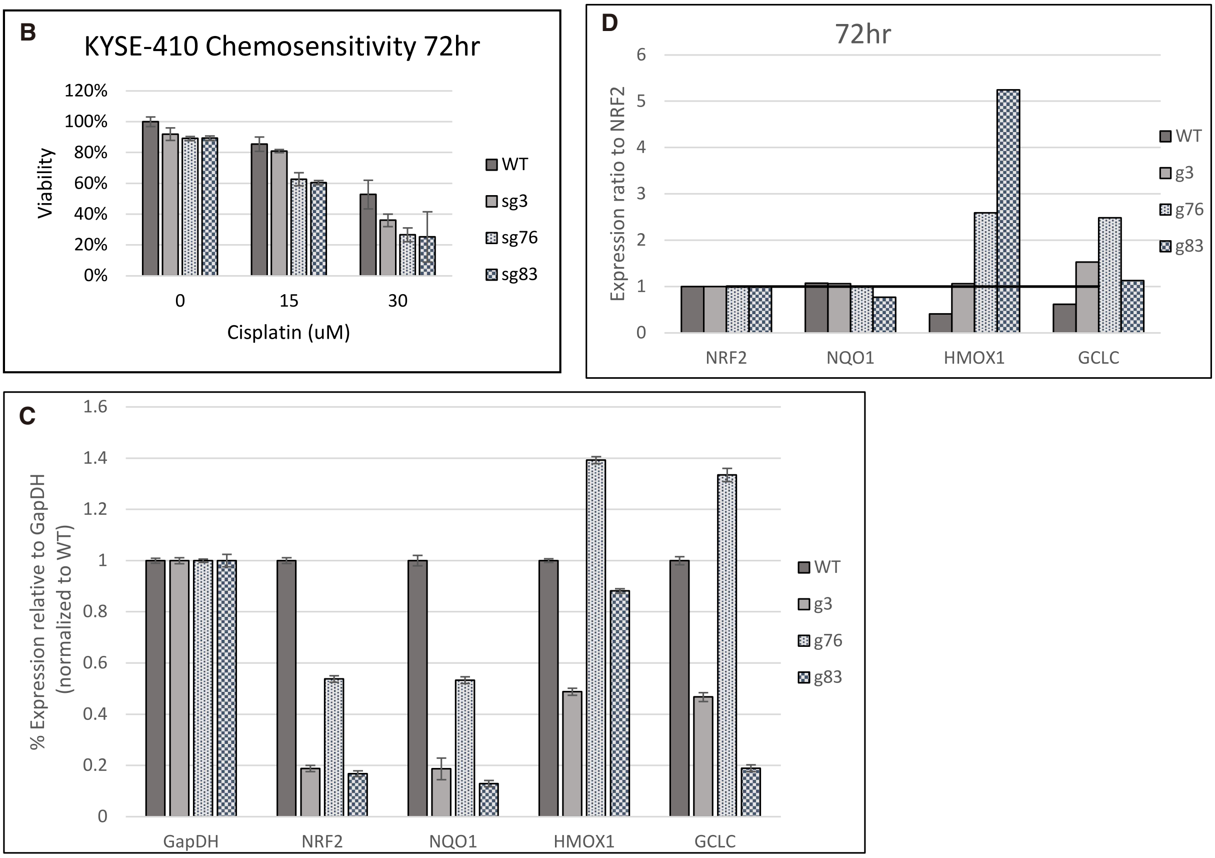

(legend on next page)

exon 2 and exon 4 are disrupted by design, and intra-genic changes are the objective of the experiment, we asked if exon skipping could account for the differential response in targeting exon 2 versus exon 4. Figure 5A represents the potential exon-skipping outcomes. For both the FaDu and the KYSE-410 cells, at 72 h post-transfection, RNA was converted to cDNA followed by PCR amplification of the NRF2 cDNA (798 bp) spanning the 5′ UTR through exon 5. The results in Figure 5B show that exon skipping is occurring, predominantly when sg3 and sg76 are used to target exon 2 and exon 4, respectively. Full-length mRNA (WT.) shows a defined band at the expected size of 798 bp. But both cell lines targeted with sg3 and sg76 present a second lower band indicative of an exon skipping event. The 798-bp band is the full transcript; the lower bands for sg3 and sg76 are 531 bp and 606 bp, respectively, corresponding to the removal of exon 2 (267 bp) and exon 4 (192 bp). In the case of exon 4 sg83-targeted KYSE-410 and FaDu cells, some level of exon skipping activity is detected, but at a much lower rate than with sg3 or sg76. To further analyze these results (Figure 5C), the cDNA products were sequenced using Sanger sequencing and aligned with the reference cDNA sequence. They were also visualized using DECODR in attempts to characterize the splicing events.[48] Similar to the genomic profile observed in the bulk-edited populations, various indels were identified in the full-length transcript, as well as larger deletions in all three target sites, which indicate exon skipping events. In the FaDu cell line (Figure S3), the sg3 targeting resulted in a 266-bp deletion (exon 2 skipping), while the sg76 and sg83 targeting resulted in deletions of 192 and 193 bp, respectively (exon 4 skipping). In the KYSE410 cell line, similar results were observed, with the addition of exon 2 and 3 skipping in the sg3-targeted cells, 267- and 247-bp deletions (Figure S4). The combined skipping of exon 3 and 4 was undetectable in the sequencing results. We understand this is due to a limitation in the sensitivity of the methods employed to analyze the Sanger sequencing results.

Sustainability of the genetic and functional disruption of NRF2

Next, we assessed if the targeted cell population maintained functional disruption of NRF2 over time. We followed the same experimental workflow, but extended cell growth for 13 days (Figure 6A). At this time, cells were collected for gDNA sequencing and RNA isolation, while the remaining cells were plated and analyzed for chemosensitivity. After 2 weeks, the exon 2 (sg3)-targeted population of cells maintained 73.45% editing (72 h: 85.5%) (Figure 6B). However, the percentage of exon 4-targeted cell populations bearing edited cells diminished slowly as a population of unedited cells expanded steadily. Exon 4 sg76 exhibited 49.30% (72 h: 87%) and sg83 exhibited 40.95% (72 h: 86%) (see Figure 6B). Indel profiles are presented in Figure S5. Interestingly, despite the reduction of indels in the cell population, the cells continued to exhibit hypersensitivity to cisplatin, even as the unedited cell population grew. Exon 4-targeted cell population still sustained higher sensitivity than the exon 2-targeted cell population (Figure 6C). To ensure the observed results were in fact due to the disruption of NRF2 and not a long-term effect of the transfection or culture conditions on the cells, we followed the same experimental workflow on cells transfected with a non-targeting scrambled guide. At both 72 h and 2 weeks, the scrambled gRNA cells maintained the same level of viability as the WT untransfected cells (Figure S6). From a genomics standpoint, there was a reduction in the total indel percent after 2 weeks; however, the percentage of cells bearing frameshift mutations within those populations remained the same (Figure 6D). Given the sustained chemosensitivity despite the change in the amount of edited cells, we assessed the status of NRF2 function on its downstream genes *NQO1*, *HMOX1*, and *GCLC* via qPCR (see Figures 6E and 6F). As seen in the 72-h analysis (Figure 3C), exon 4 sg83 results in lower expression of *NQO1* and *GCLC* compared to sg3 and sg76. In the case of exon 2 sg3, the opposite effect is seen, where there is an increase in the expression of *NQO1, HMOX1*, and *GCLC*. These results could be attributed to the effects captured over time of disrupting the two different functional domains of NRF2 acting in opposite functions. Exon skipping was also examined with no substantial changes in the results observed at 72 h (Figure 6G). These results confirm that the functional outcomes are sustainable and could provide some insight into the importance of the type of indels that are most effective in producing those outcomes (Indel Code).

DISCUSSION

The overall incidence of HNC continues to rise, attributed to lifestyle habits (especially in Asia-Pacific regions) and the increasing rate of human papillomavirus infection in the United States and Europe.[2,49] Despite the advancement in cancer care, the development of drug resistance remains a major obstacle in reducing patient suffering. We are developing a CRISPR-Cas gene editing approach to disable genes promoting or enabling resistance to standard care. This type of platform is reflective of the broad-based applications surrounding human gene editing directed by programmable nucleases, including CRISPR-Cas. We take advantage of the inherent activity of CRISPR-Cas in the prokaryotic environment and apply it to the genetic disruption of the *NRF2* gene.[50] This gene regulates the response to

Figure 4. Genetic disruption of *NRF2* in KYSE-410 cells

(A) Genomic analyses of *NRF2* after CRISPR targeting. Genomic DNA from KYSE-410 cells was isolated and amplified across exon 2 and exon 4 of the *NRF2* gene. Amplicon was NGS sequenced and analyzed for indels at the CRISPR target site. Raw sequence files were aligned using the software program, CRISPResso2, to display the *NRF2* allele-specific indel pattern and frameshift percent of the targeted outcomes. (B) Chemosensitivity testing in response to *NRF2* targeting. Chemosensitivity was measured via CellTiter-Glo 2.0 Assay. Targeted cells were treated with increasing concentrations of cisplatin for 72 h and then evaluated for cell viability. The average relative viability of cells normalized to the untreated WT was graphed. The error bars represent the coefficient of variance. (C) qPCR gene expression analysis. RNA was isolated from targeted cells and converted to cDNA. Relative gene expression of *NRF2*, *NQO1*, *HMOX1*, and *GCLC* was measured through qPCR and analyzed with the Pfaffl method. Relative gene expression normalized to the WT cells was graphed. The error bars represent the coefficient of variance.(D) Ratio of *NRF2* expression to downstream targets. Values from (C) were divided by *NRF2* expression values for each condition to show the ratio of *NRF2* to *NQO1*, *HMOX1*, and *GCLC* when *NRF2* expression is reduced.

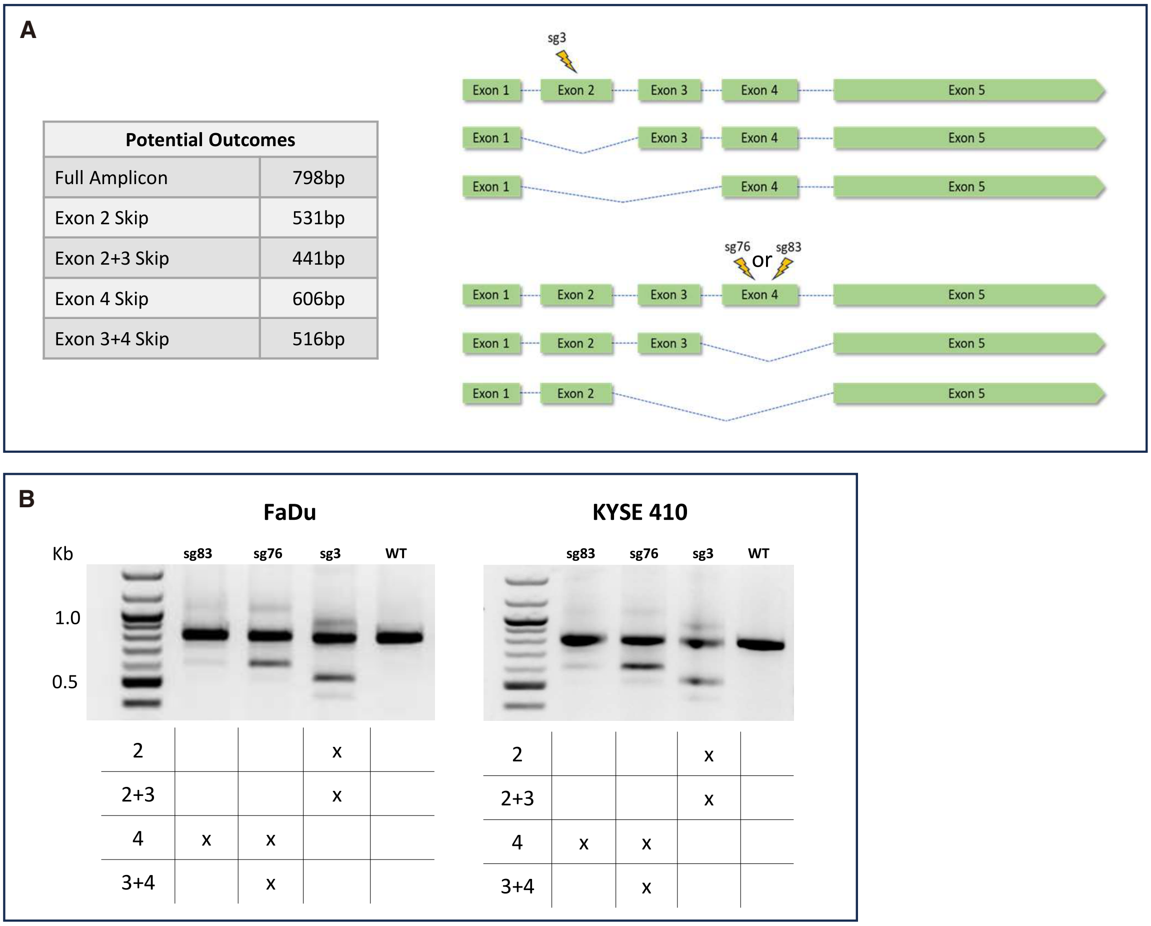

C

	FaDu	KYSE
**sgRNA**	**Size of seq product deletion**	
sg3	-266	-247, -267
sg76	-192, -193	-192, -193
sg83	-192, -193	-192, -193

Figure 5. Exon-skipping NRF2 gene after CRISPR-Cas9 disruption
cDNA from cells targeted with a CRISPR guide RNA in exon 2 or exon 4 was collected at 72 h post-transfection and was used as the template for PCR amplification with primers that spanned from the 5′ UTR region through exon 5 (798 bp) of the NRF2 gene. (A) A diagram to show targeting of exon 2 and exon 4, and the resulting exon-skipped variants. (B) cDNA gels for each sgRNA targeting condition with exon-skipped variants listed beneath. (C). NRF2 cDNA was analyzed using DECODR, and the deletion size output representing skipped sequence for each condition in (B) is listed in the table.

various environmental stresses, including treatment with anti-cancer drugs. This transcription factor can accelerate the growth of transformed or cancerous cells and enhance migration and invasion, even inflammation.[51] The tumor-promoting activity has been associated with high expression of the *NRF2* gene as a consequence of disruptions to the NRF2-KEAP1 interaction by mutations in both genes, epigenetic changes affecting *NRF2* expression, and direct transcriptional activation of Nrf2 from oncogenes.[22–24,52,53] NRF2 has

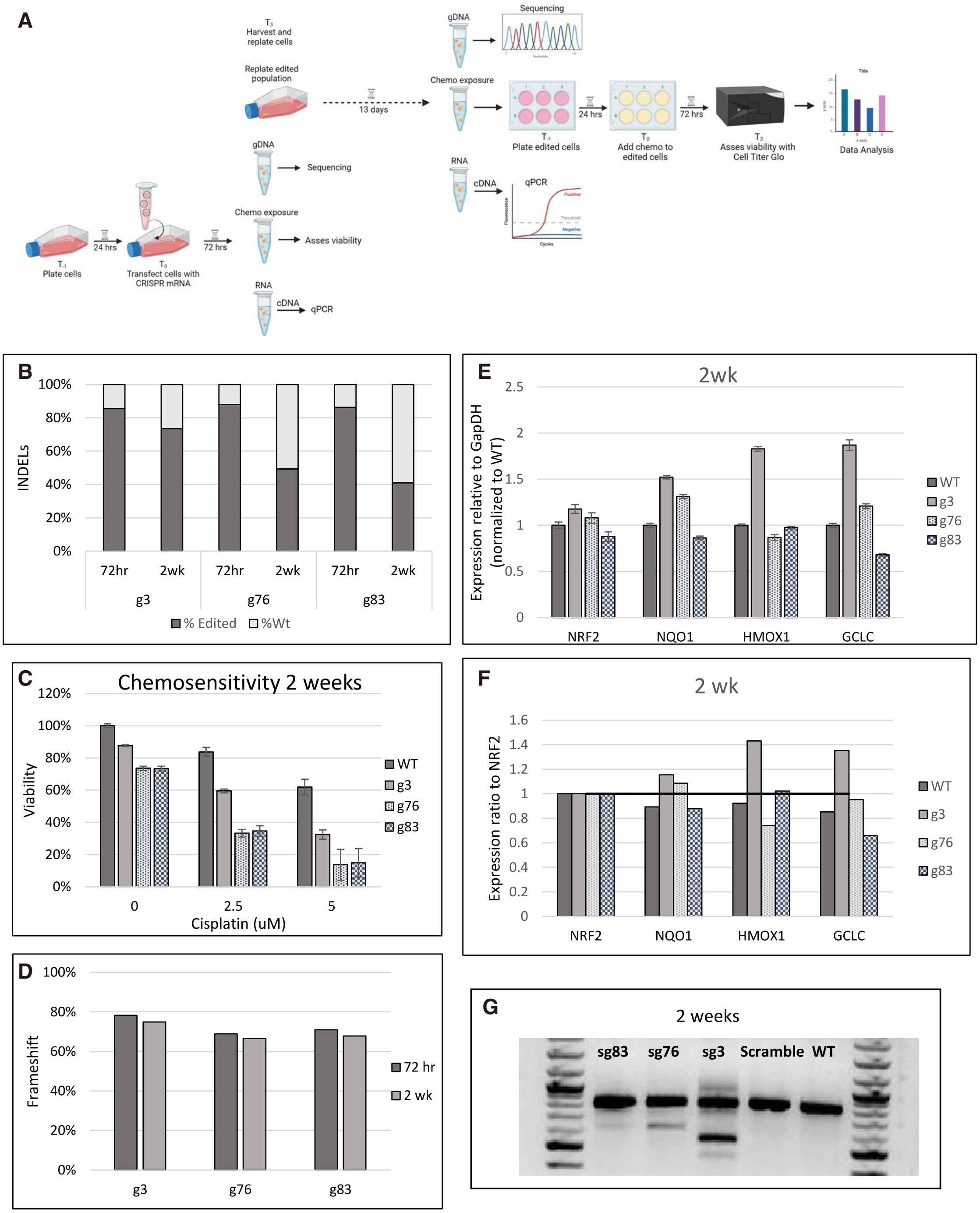

Figure 6. Sustained genetic and functional disruption of NRF2 over time

(A) Experimental workflow to assess editing outcomes in NRF2 2 weeks after transfection. (B and D) Genomic analyses of NRF2 2 weeks post-CRISPR/Cas9 targeting. Genomic DNA from FaDu cells was isolated and amplified across exon 2 and exon 4 of the *NRF2* gene 13 days after transfection. Amplicon was NGS sequenced and analyzed for indels at the CRISPR target site. Raw sequence files were aligned using the software program, CRISPResso2, to display the *NRF2* allele-specific indel pattern and frameshift percent of the targeted outcomes. (C) Sustained chemosensitivity testing 2 weeks after CRISPR-Cas9 targeting of NRF2. Chemosensitivity was measured via CellTiter-Glo 2.0 Assay. Targeted cells that were maintained in culture for 2 weeks post-transfection were treated with increasing concentrations of cisplatin for 72 h and then evaluated for cell viability. The average relative viability of cells normalized to the untreated WT was graphed. The error bars represent the coefficient of variance. (E) qPCR

(legend continued on next page)

been elucidated as an important target for cancer treatment but had been deemed undruggable.[24,54] Our laboratory has overcome the undruggable nature of NRF2 by utilizing CRISPR-Cas9 to disrupt the gene. We have aimed to demonstrate the reversal of drug resistance, with the potential of gaining sensitivity to other cancer therapies.

In this paper, we describe the genetic and functional consequences of NRF2 disruption at two distinct exon-targeting sites in HNC and esophageal cancer cells. Our results indicate that exon 2 and exon 4 are suitable targets for genetic disruption as the level of indels produced through the action of CRISPR-Cas exceeds 70%. More importantly, the indel profile revealed a prevalence of frameshift mutations, the critical guidepost to the effectiveness of true knockout in human cells. We believe there is a hierarchy of productive mutations within the indel population.[28] This hierarchy reveals the probability of effective gene knockout, not only from the genotypic standpoint but also, more importantly, from the functional standpoint.

Previously, we were able to effectively disrupt NRF2 at multiple exons in adenocarcinoma and squamous cell carcinoma of the lung, initially by genetically engineering NRF2 knockout cells, then implanting in animals, and subsequently by disrupting the gene in cells directly.[26,32] Now, we have expanded the platform of NRF2 knockout and have examined, in much greater detail, the impact that such a knockout has on the behavior of cancer cells. Productive exon 2 disruption in FaDu cells is quite efficient, often exceeding 70%, yet even with this high level of indel formation, the restoration of chemosensitivity (cisplatin and 5-FU) was modest at best. Specifically, sg3, while being effective at genetic disruption, was ineffective in producing the desired functional outcome, while conserving protein expression at 70%. In contrast, exon 4 disruption by sg76 and sg83 provided both significant genetic disruption and the desired functional outcome with a substantial knockdown of the NRF2 protein. There is a substantial increase in chemosensitivity at low doses, when sg76 and sg83 execute the knockout of NRF2. As seen in Figure 1, these two exons encode significantly different protein domains. Exon 2 encodes a section of the protein that enables binding of the sister protein KEAP1, whereas exon 4 encodes the protein domain involved in transactivation, a more critical function of NRF2 in its role as a transcription factor. The impact of exon 4 disruption can also be seen in the lower level of *NQO1* and *GCLC* expression, as predicted. These data reinforce the importance of evaluating multiple knockout sites when designing gRNAs to achieve phenotypic change, particularly if the endpoint, as in our case, has clinical relevance. Along with achieving the therapeutic outcome we seek by targeting exon 4 in NRF2, both sgRNA 76 and 83 resulted in less predicted off-target sites compared to sg3 when assessed by the *in silico* prediction tool Cas-OFFinder.[36] Although we recognize that a more thorough detection of potential off-targets and subsequent validation needs to be performed to assess the off-target profiles more comprehensively, we believe exon 4 targeting with sg83 in conjunction with a high-fidelity Cas9 variant and a transient delivery method such as LNP (lipid nano particle) will suffice regulatory requirements.

We sought to expand the utility of this approach in a different cancer subtype and retested these gRNAs in a cell system derived from an esophageal tumor; in general, the pattern of results was the same. Exon 4 disruption was more effective in restoring chemosensitivity, even though the levels of indel formation were similar to those achieved in exon 2. Increasing the dosage of chemotherapy significantly (Figure 4B) increased sensitivity as a function of disruption of exon 2 by sg3, consistent with the general concept that NRF2 is a key player in the establishment of chemoresistance in tumor cells.

So, why is targeting exon 4 more effective than exon 2 in generating the expected desired phenotypic and functional outcomes? While we had already offered one explanation centered on the importance of the functional protein domains correlating to the desired outcomes, CRISPR-directed gene editing has also been shown to induce a series of molecular rearrangements or re-designations.[55] These changes can take the form of nucleotide exchange in unwarranted sites,[56] chromosomal translocation,[57] and exon skipping.[32] The last of these molecular gymnastics was a centerpiece of earlier work on NRF2 in lung cancer.[32] We discovered that exon skipping occurred in both exons in adenocarcinoma cells and, more importantly, influenced the degree of response to chemotherapy. While chemosensitivity was restored in cancer cells that harbored exon skipping, our evidence suggests that this rearrangement influences functional outcome. Here, all three sgRNAs tested induce exon skipping in both HNC and esophageal cancer cells. In the case of sg3, it is possible that the degree of exon skipping reduces the effectiveness of CRISPR-directed gene editing of *NRF2* in the restoration of chemosensitivity. The loss of the Neh2 domain encoded within exon 2 disables the binding of KEAP1, interfering in the degradation process of NRF2. This would explain why 80%–90% genetic disruption only reduced protein by 30%. As for targeting exon 4, sg76 and sg83 disrupt the junction site between the Neh4 and Neh5 domains, which are responsible for NRF2 transcriptional factor activity. While we cannot detect splice variant isoforms in the bulk edited population, previous clonal work suggests that exon-skipped RNA variants do result in truncated protein products.[32] The loss of these sites is detrimental to the protein activity. When comparing the expression

gene expression analysis. RNA was isolated from edited cells at the 2-week collection time point, converted to cDNA and transcript levels of *NRF2*, *NQO1*, *HMOX1* and *GCLC* were measured by qPCR. The error bars represent the coefficient of variance. (F) Ratio of NRF2 expression to downstream targets. Values from (E) were divided by NRF2 expression values for each condition to show the ratio of *NRF2* to *NQO*1, *HMOX1*, and *GCLC* when *NRF2* expression is reduced 2 weeks post-targeting. (G) Exon skipping 2 weeks post-targeting. cDNA from cells targeted with a CRISPR guide RNA in exon 2 or exon 4 were collected at 2 weeks post-transfection and were used as the template for PCR amplification with primers that spanned from the 5′ UTR region through exon 5 (798 bp) of the NRF2 gene. cDNA gel for each condition 2 weeks post-targeting is shown.

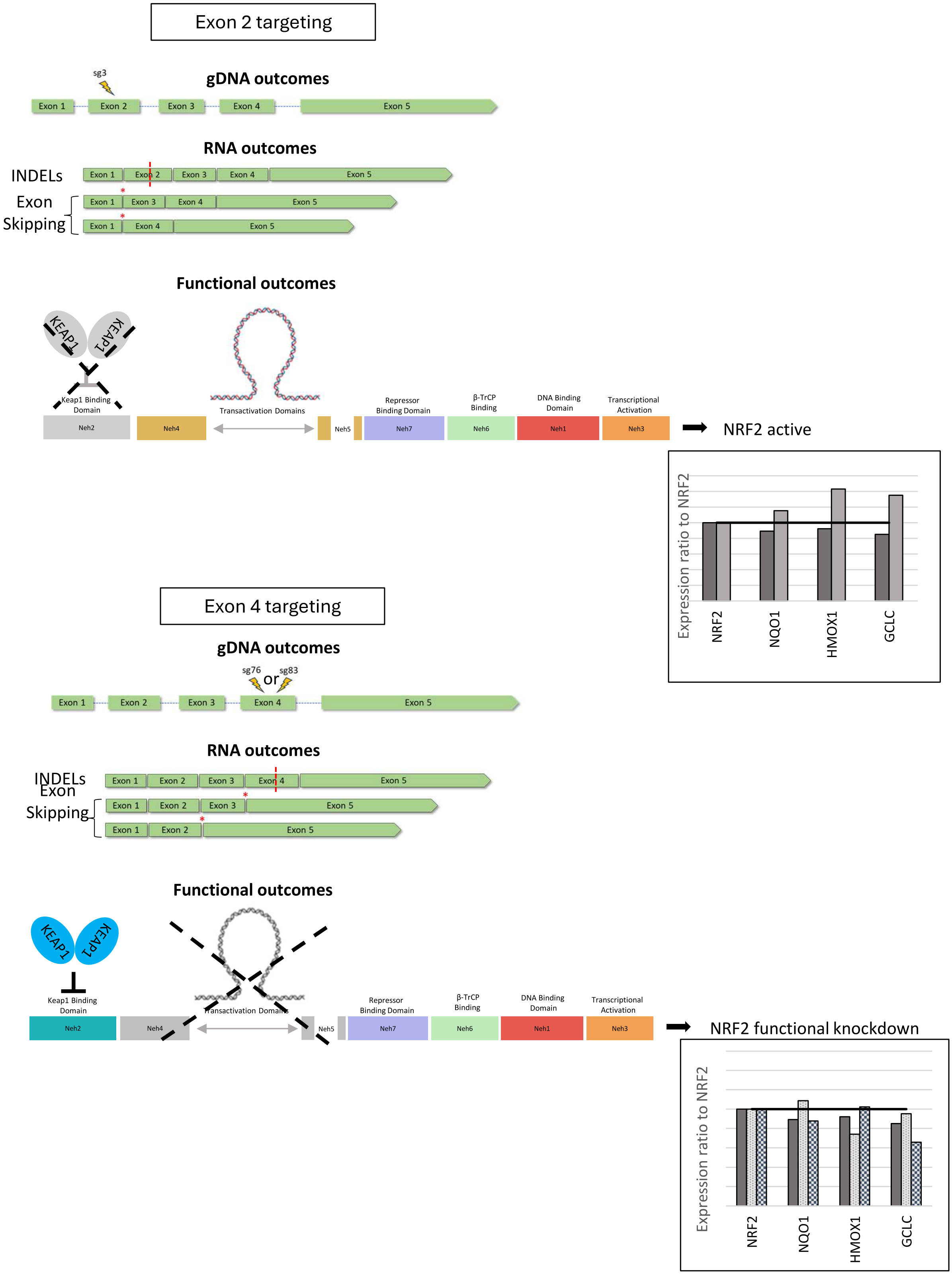

(legend on next page)

ratio between *NRF2* and downstream effectors, targeting in exon 2 results in higher expression than targeting in exon 4, despite similar genetic knockout profiles. Once again, the pure genetic profiles of gene editing in human cells, especially those with tantalizingly high levels of activity, are dependent on which site is disrupted, and may not be predictive of functional outcome (Figure 7).

Despite attempts at disabling NRF2 in all tumor cells, the likelihood of achieving this endpoint is and will be low. While our levels of genotypic knockout are impressive, often achieving greater than 70%, cells that escape genetic alteration and those that are nonproductively edited likely continue to proliferate and overtake the population, an observation in Figure 6C. Our data suggest that over time, the percentage of gene-edited cells of all profiles diminishes as the total cell population expands. Interestingly, the percentage of frameshift mutation remained steady even as the overall population expanded. We hypothesize that indel distribution is a factor in the shift from high editing to WT genotype. Cancer cell lines have variable copy numbers, so it is plausible that cells with multiple indels die, while cells with WT alleles carry indels but compensate and survive. While these cells may survive, they cannot mount a stress response and, therefore, maintain chemosensitivity. This is a good sign for therapeutic application since we know from the indel code[28] that these types of disruption are more effective in changing phenotype. However, the effect of the frameshift population is evident in the sustained chemosensitivity in FaDu cells genetically modified by sg3, sg76, and sg83. At this later time, exon 4 edited by sg76 and sg83 sustained higher chemosensitivity despite having lower edited cells compared to exon 2 sg3.

We will continue to explore the hierarchy of CRISPR-directed gene editing in human cells with an emphasis on improving treatment outcomes. While the efficiency of genetic knockout using CRISPR-Cas complexes is not in question, the functional readout and effectiveness of these knockouts as written in the underlying sequences remain problematic. We believe that utilizing gene editing as a form of genetic medicine in combination with standard of care will overcome resistance at the genetic level, sustaining the effectiveness of anticancer drugs and treatments. Our results can potentially codify a platform technology that targets and disables genes promoting resistance to standard chemotherapy in a wider array of solid tumors.

MATERIAL AND METHODS

Cell line and culture conditions

Human esophageal squamous cell carcinoma KYSE-410 cells and hypopharyngeal squamous cell carcinoma FaDu cells were purchased from MilliporeSigma (Burlington, MA, USA) and ATCC (Manassas, VA, USA), respectively. Cells were thawed, according to the manufacturer's protocol. KYSE-410 cells (KYSE, hereafter) were cultured in RPMI medium augmented with 2 mM glutamine and 10% fetal bovine serum (FBS) and grown at 37°C in 5% CO_2. FaDu cells were grown in Eagle's minimum essential medium supplemented with 10% FBS and grown at 37°C in 5% CO_2. Cell lines were evaluated for *Mycoplasma* upon thawing and before use in experiments using the MycoScope PCR Mycoplasma detection kit (AMSBIO, Cambridge, MA).

CRISPR-Cas9 design

The *NRF2* gene-coding sequence(NCBI: NC_000002, Gene ID: 4780) was entered into SnapGene, and the following gRNA was selected for targeting exon 2: (sg3) 5′-TATTTGACTTCAGTCAGCGA-3′, as well as two gRNAs targeting exon 4: (sg76) 5′-GTCACTTGTTCCTGATATTCCCGG-3′ and (sg83) 5′-GTAGCCCCTGTTGATTTAGACGG-3′. Based on the gRNA design, synthetic single gRNAs were ordered from Synthego (Menlo Park, California, USA). CleanCap Cas9 mRNA (5moU) was ordered from TriLink Biotechnologies (San Diego, CA, USA). *In silico* off-target analysis was performed using Cas-OFFinder[36] (http://www.rgenome.net/cas-offinder/) for sg3, sg76, and sg83, allowing up to four mismatches and recognizing both NAG and NGG PAMs (GRCh38: Genome Reference Consortium human build 38; MM: mismatch; OnT: On-target).

Lipofectamine MessengerMAX transfection

Either 1 million cells were seeded to a 75-cm^2 tissue culture flask or 2.5 million cells were seeded to a 175 cm^2-tissue culture flask 24 h prior to transfection and allowed to reach 60%–80% confluency. On the day of the transfection, cells were treated with Lipofectamine MessengerMAX reagent (Thermo Fisher Scientific, Waltham, MA) bearing 10 or 25 μg of Cas9mRNA and 21.89 or 55 μg of sgRNA, suspended in Opti-MEM. 39 or 97.5 μL of MessengerMAX lipofectamine reagent was diluted in 1.5 or 3.75 mL OPTI-MEM medium, vortexed twice, and then incubated for 10 minutes at room temperature, during which time the culture media was replaced with 15 or 25 mL of fresh media, as appropriate to the cell line. A total of 31.89 or 80 μg of total RNA was then added to the diluted Lipofectamine, consisting of 10 or 25 μg Cas9 mRNA and 10 or 25 μg sgRNA. The suspension was incubated at room temperature for an additional 5 min before being added, in full, to the tissue culture flask, which was then swirled gently to disperse the reagent. Treated cells were incubated for 72 h at 37°C prior to being assayed.

Next-generation sequencing gene editing analysis

Targeted amplicon libraries were prepared using Illumina's 16S metagenomic sequencing library preparation protocol for MiSeq. Genomic

Figure 7. Mechanism model

The selection of the target site by gene editing influences the functional outcomes and is not solely dependent on high levels of activity. Exon skipping rearrangement influences the functional outcome. The loss of the Neh2 domain encoded within exon 2 disables the binding of KEAP1 keeping NRF2 protein from degrading and keeping its transcription factor activity. Exon 4 targeting disrupts the junction site between the Neh4 and Neh5 domains responsible for NRF2 transcriptional factor activity rendering them inactive.

DNA was extracted from cells using the Qiagen DNeasy Blood & Tissue kit (Qiagen, Hilden, Germany) or QuickExtract reagent (Biosearch Technologies). The region surrounding the CRISPR target site for exon 2 (398 bp) and exon 4 (402 bp) of the NRF2 gene was amplified using the 2X Phusion Flash PCR master mix (Thermo Fisher Scientific, Waltham, MA). Post-amplification, samples were cleaned using AMPure beads and DNA concentration was measured using the Qubit assay (Thermo Fisher Scientific, Waltham, MA). Samples were run on TapeStation (Agilent Technologies, Santa Clara, CA) to verify the presence of the desired amplicon and indexed using the IDT for Illumina DNA/RNA UD indexes set A (Illumina, San Diego, CA). Samples were again quantified using Qubit, and average library size was calculated using the TapeStation amplicon size. Once pooled to the loading concentration, the libraries were sequenced using the MiSeq Reagent kit v2 (Illumina, San Diego, CA). Only the data that passed data QC in Sequencing Analysis Viewer were analyzed using CRISPResso2 (crispresso2.pinellolab.org) to understand gene editing efficiency and frameshift analysis.

Cell viability

Transfected and WT KYSE and FaDu cells were plated in 24-well plates (n = 4) at 50,000 cells per well and incubated for 24 h. The cells were then treated with: 0, 2.5, 5, 15, or 30 μM cisplatin (a gift from ChristianaCare Pharmacy) or with 100 or 300 μM 5-FU (Selleck, Houston, TX) for 3 days. Cell viability after drug exposure was evaluated using the CellTiter-Glo 2.0 Cell Viability Assay (Promega, Madison, WI). CellTiter-Glo reagent was added to wells at 1:1 volume with the cell suspension. The plate was covered in foil and placed on an orbital shaker for 2 min before being removed. The plate was allowed to equilibrate at room temperature for another 10 min before the luminescence of its contents was measured using an Infinite 2000 PRO microplate reader (Tecan, Männedorf, Switzerland). The data were plotted with the coefficient of variance.

Reverse-transcription PCR

Total RNA was isolated from edited cells using TRIzol reagent and PureLink RNA Mini Kit (Thermo Fisher Scientific, Waltham, MA). Reverse transcription was conducted using the Applied Biosystems High-Capacity RNA-to-cDNA kit (Thermo Fisher Scientific, Waltham, MA). The cDNA was used as the template in the qPCR amplification of GAPDH (Fwd: 5′ TCTCCTCTGACTTCAACAGCGAC3′, Rev: 5′CCCTGTTGCTGTAGCCAAATTC3′), NRF2 (Fwd: 5′TCCAAGTCCAGAAGCCAAACTGAC3′, Rev: 5′GGAGAGGATGCTGCTGAAGGAATC3′), NQO1 (Fwd: 5′GGTTTGGAGTCCCTGCCATT3′, Rev: 5′TTGCAGAGAGTACATGGAGCC3′), HMOX1 (Fwd: 5′ CTTTCAGAAGGGCCAGGTGA3′, Rev: 5′GTAGACAGGGGCGAAGACTG3′), and GCLC (Fwd: 5′GGACAAGAATACACCATCTCCA3′, Rev: 5′ ATACTGCAGGCTTGGAATGTC3′) transcripts using the SsoAdvanced Universal SYBR Green Supermix (Bio-Rad, Hercules, CA). Panel was done on a single experiment with 6 technical replicates each. Relative expression levels of mRNA were calculated using the Livak method and normalized to the control. The data were plotted with the coefficient of variance.

Exon skipping analysis

Total RNA was isolated from edited cells using TRIzol reagent and PureLink RNA Mini Kit (Thermo Fisher Scientific, Waltham, MA). Reverse transcription was conducted using the Applied Biosystems High-Capacity RNA-to-cDNA kit (Thermo Fisher Scientific, Waltham, MA). The cDNA was used as the template for PCR amplification with either Phusion Flash or Phusion High-Fidelity PCR master mix (Thermo Fisher Scientific, Waltham, MA) with primers spanning the 5′ UTR region through exon 5 (798 bp) of the NRF2 gene. PCR reactions were purified using Qiagen QIAquick PCR cleanup columns (Qiagen, Hilden, Germany) according to manufacturer's protocol, measured on a Nanodrop and equally loaded on a 1% agarose gel. Amplified cDNA was used as template in BigDye Terminator V3.1 cycle sequencing kit reactions (Applied Biosystems) for Sanger sequencing and visualized in SnapGene (snapgene.com) and DECODR[48] (Decodr.org).

Protein preparation and western blotting

Total protein was isolated from cells in RIPA buffer with a protease inhibitor cocktail (Pierce) and incubated on ice for 30 min with vortexing every 10 min. Samples were centrifuged at $14,000 \times g$ at 4°C for 15 min, and the supernatant was saved. Protein extracts were quantified using a BCA assay (Pierce), and 20 μg of protein was mixed 3:1 with Laemmli sample buffer plus 5% Beta-mercaptoethanol (Bio-Rad) and loaded onto a 4%–20% polyacrylamide pre-cast gel (Bio-Rad). Protein was transferred onto a 0.2 μM nitrocellulose blot using the Turboblot dry transfer system (Bio-Rad). The membrane was blocked in 5% milk in TBS-T for 2 h at room temperature and stained with anti-NRF2 1:1,000 (Abcam, ab62352) and anti-GAPDH 1:5,000 (Cell Signaling Technology, 97166). Blots were washed 3× at room temperature in TBS-T and stained with secondary antibodies conjugated to HRP (horseradish peroxidase) 1:10,000 (Abcam, ab205718 or Thermo Fisher, PI31430). Blots were imaged using the Pierce Femto western blotting substrate (Pierce). Bands were quantified using the FIJI gel analysis tool package (https://imagej.net/ij/).

DATA AND CODE AVAILABILITY

We are willing to share any data reported in this paper after it is published, upon a request from the lead contact. This paper does not report any original code. Any additional information required to reanalyze the data reported in this paper is available upon request from the lead contact.

ACKNOWLEDGMENTS

This project was supported by grants from the National Institute of General Medical Sciences (P20 748GM103446 and P20 GM109021), and from the Carol A Ammon Foundation, United States and Lisa Dean Moseley Foundation, United States. The work was also funded by grants from CorriXR Therapeutics. This content is solely the responsibility of the authors and does not necessarily represent the official views of the NIH.

AUTHOR CONTRIBUTIONS

N.R.-T. contributed to the conceptualization, formal analysis, investigation, methodology, project administration, resources, supervision, validation, visualization, writing – original draft and review/editing. L.E.S. contributed to formal analysis, investigation, methodology, supervision, validation, visualization, writing – original draft and review/editing. J.A.R. contributed to formal analysis, investigation,

methodology, supervision, validation, visualization, and writing – original draft. G.A. contributed to the investigation, validation, and visualization. K.B. contributed to conceptualization, methodology, supervision, and validation. P.B. contributed to formal analysis, conceptualization, methodology, supervision, validation, and review/editing. E.B.K. contributed to conceptualization, funding acquisition, resources, supervision, writing – original draft and review/editing.

DECLARATION OF INTERESTS

E.B.K. is a founder of the company CorriXR Therapeutics and serves as a fractional CEO. N.R.-T., K.B., and P.B. hold shares in CorriXR Therapeutics. N.R.-T., K.B., and P.B. are consultants to CorriXR Therapeutics. E.B.K., P.B., K.B., and N.R.-T. have patents US12203070B2 (awarded) and 130949-01800 (pending) related to this work.

SUPPLEMENTAL INFORMATION

Supplemental information can be found online at https://doi.org/10.1016/j.omton.2025.201122.

REFERENCES

1. Sung, H., Ferlay, J., Siegel, R.L., Laversanne, M., Soerjomataram, I., Jemal, A., and Bray, F. (2021). Global Cancer Statistics 2020: GLOBOCAN Estimates of Incidence and Mortality Worldwide for 36 Cancers in 185 Countries. CA Cancer J. Clin. *71*, 209–249. https://doi.org/10.3322/CAAC.21660.
2. Gormley, M., Creaney, G., Schache, A., Ingarfield, K., and Conway, D.I. (2022). Reviewing the epidemiology of head and neck cancer: definitions, trends and risk factors. Br. Dent. J. *233*, 780–786. https://doi.org/10.1038/S41415-022-5166-X.
3. Johnson, D.E., Burtness, B., Leemans, C.R., Lui, V.W.Y., Bauman, J.E., and Grandis, J.R. (2020). Head and neck squamous cell carcinoma. Nat. Rev. Dis. Primers *6*, 92. https://doi.org/10.1038/s41572-020-00224-3.
4. Sung, H., Ferlay, J., Siegel, R.L., Laversanne, M., Soerjomataram, I., Jemal, A., and Bray, F. (2021). Global Cancer Statistics 2020: GLOBOCAN Estimates of Incidence and Mortality Worldwide for 36 Cancers in 185 Countries. CA Cancer J. Clin. *71*, 209–249. https://doi.org/10.3322/caac.21660.
5. Head and Neck Cancer Statistics Cancer.Net. https://www.cancer.net/cancer-types/head-and-neck-cancer/statistics.
6. Siegel, R.L., Miller, K.D., Wagle, N.S., Jemal, A., Ahmedin, |, Dvm, J., and Siegel, R.L. (2023). Cancer statistics, 2023. CA Cancer J. Clin. *73*, 17–48. https://doi.org/10.3322/caac.21763.
7. Johnson, D.E., Burtness, B., Leemans, C.R., Lui, V.W.Y., Bauman, J.E., and Grandis, J.R. (2020). Head and neck squamous cell carcinoma. Nat. Rev. Dis. Primers *6*, 92. https://doi.org/10.1038/s41572-020-00224-3.
8. Wise-Draper, T.M., Bahig, H., Tonneau, M., Karivedu, V., and Burtness, B. (2022). Current Therapy for Metastatic Head and Neck Cancer: Evidence, Opportunities, and Challenges (American Society of Clinical Oncology Educational Book), pp. 527–540. https://doi.org/10.1200/edbk_350442.
9. Fuereder, T. (2022). Essential News of Current Guidelines: Head and Neck Squamous Cell Carcinoma (Springer). https://doi.org/10.1007/s12254-022-00842-5.
10. Picon, H., and Guddati, A.K. (2020). Mechanisms of resistance in head and neck cancer. Am. J. Cancer Res. *10*, 2742–2751.
11. Housman, G., Byler, S., Heerboth, S., Lapinska, K., Longacre, M., Snyder, N., and Sarkar, S. (2014). Drug Resistance in Cancer: An Overview. Cancers (Basel) *6*, 1769–1792. https://doi.org/10.3390/cancers6031769.
12. Mansoori, B., Mohammadi, A., Davudian, S., Shirjang, S., and Baradaran, B. (2017). The Different Mechanisms of Cancer Drug Resistance: A Brief Review. Adv. Pharm. Bull. *7*, 339–348. https://doi.org/10.15171/apb.2017.041.
13. Min, H.-Y., and Lee, H.-Y. (2021). Mechanisms of resistance to chemotherapy in non-small cell lung cancer. Arch Pharm. Res. (Seoul) *44*, 146–164. https://doi.org/10.1007/s12272-021-01312-y.
14. Sánchez-Ortega, M., Carrera, A.C., and Garrido, A. (2021). Role of NRF2 in Lung Cancer. Cells *10*, 1879. https://doi.org/10.3390/cells10081879.
15. Zhao, J., Lin, X., Meng, D., Zeng, L., Zhuang, R., Huang, S., Lv, W., and Hu, J. (2020). Nrf2 Mediates Metabolic Reprogramming in Non-Small Cell Lung Cancer. Front. Oncol. *10*, 578315. https://doi.org/10.3389/fonc.2020.578315.
16. Matsuoka, Y., Yoshida, R., Kawahara, K., Sakata, J., Arita, H., Nkashima, H., Takahashi, N., Hirayama, M., Nagata, M., Hirosue, A., et al. (2022). The antioxidative stress regulator Nrf2 potentiates radioresistance of oral squamous cell carcinoma accompanied with metabolic modulation. Lab. Invest. *102*, 896–907. https://doi.org/10.1038/s41374-022-00776-w.
17. Feng, L., Zhao, K., Sun, L., Yin, X., Zhang, J., Liu, C., and Li, B. (2021). SLC7A11 regulated by NRF2 modulates esophageal squamous cell carcinoma radiosensitivity by inhibiting ferroptosis. J. Transl. Med. *19*, 367. https://doi.org/10.1186/s12967-021-03042-7.
18. Shibata, T., Kokubu, A., Saito, S., Narisawa-Saito, M., Sasaki, H., Aoyagi, K., Yoshimatsu, Y., Tachimori, Y., Kushima, R., Kiyono, T., and Yamamoto, M. (2011). NRF2 mutation confers malignant potential and resistance to chemoradiation therapy in advanced esophageal squamous cancer. Neoplasia *13*, 864–873. https://doi.org/10.1593/neo.11750.
19. Baird, L., and Yamamoto, M. (2020). The Molecular Mechanisms Regulating the KEAP1-NRF2 Pathway. Mol. Cell Biol. *40*, e00099-20. https://doi.org/10.1128/MCB.00099-20.
20. Hayes, J.D., and McMahon, M. (2009). NRF2 and KEAP1 mutations: permanent activation of an adaptive response in cancer. Trends Biochem. Sci. *34*, 176–188. https://doi.org/10.1016/j.tibs.2008.12.008.
21. Menegon, S., Columbano, A., and Giordano, S. (2016). The Dual Roles of NRF2 in Cancer. Trends Mol. Med. *22*, 578–593.
22. Wang, X.J., Sun, Z., Villeneuve, N.F., Zhang, S., Zhao, F., Li, Y., Chen, W., Yi, X., Zheng, W., Wondrak, G.T., et al. (2008). Nrf2 enhances resistance of cancer cells to chemotherapeutic drugs, the dark side of Nrf2. Carcinogenesis *29*, 1235–1243. https://doi.org/10.1093/carcin/bgn095.
23. Cloer, E.W., Goldfarb, D., Schrank, T.P., Weissman, B.E., and Major, M.B. (2019). NRF2 activation in cancer: from DNA to protein. Cancer Res. *79*, 889–898.
24. Lin, L., Wu, Q., Lu, F., Lei, J., Zhou, Y., Liu, Y., Zhu, N., Yu, Y., Ning, Z., She, T., et al. (2023). Nrf2 signaling pathway: current status and potential therapeutic targetable role in human cancers. Front. Oncol. *13*, 1184079. https://doi.org/10.3389/fonc.2023.1184079.
25. Morgenstern, C., Lastres-Becker, I., Demirdöğen, B.C., Costa, V.M., Daiber, A., Foresti, R., Motterlini, R., Kalyoncu, S., Arioz, B.I., Genc, S., et al. (2024). Biomarkers of NRF2 signalling: Current status and future challenges. Redox Biol. *72*, 103134. https://doi.org/10.1016/J.REDOX.2024.103134.
26. Bialk, P., Wang, Y., Banas, K., and Kmiec, E.B. (2018). Functional Gene Knockout of NRF2 Increases Chemosensitivity of Human Lung Cancer A549 Cells in Vitro and in a Xenograft Mouse Model. Mol. Ther. Oncolytics *11*, 75–89. https://doi.org/10.1016/j.omto.2018.10.002.
27. Rivera-Torres, N., Bialk, P., and Kmiec, E.B. (2023). CRISPR-Directed Gene Editing as a Method to Reduce Chemoresistance in Lung Cancer Cells. Methods Mol. Biol. *2660*, 263–271. https://doi.org/10.1007/978-1-0716-3163-8_18.
28. Banas, K.H., Bialk, P.A., Rivera-Torres, N., Owens, K., Reiner, T.N., Pisarcik, K.M., Haas, N., Gielda, E., Khan, K., Narra, K.P., and Kmiec, E.B. (2025). Functional characterization of tumor-specific CRISPR-directed gene editing as a combinatorial therapy for the treatment of solid tumors. Mol. Ther. Oncol. *33*, 201079. https://doi.org/10.1016/J.OMTON.2025.201079.
29. Singh, A., Misra, V., Thimmulappa, R.K., Lee, H., Ames, S., Hoque, M.O., Herman, J. G., Baylin, S.B., Sidransky, D., Gabrielson, E., et al. (2006). Dysfunctional KEAP1-NRF2 interaction in non-small-cell lung cancer. PLoS Med. *3*, e420–e1876. https://doi.org/10.1371/journal.pmed.0030420.
30. Solis, L.M., Behrens, C., Dong, W., Suraokar, M., Ozburn, N.C., Moran, C.A., Corvalan, A.H., Biswal, S., Swisher, S.G., Bekele, B.N., et al. (2010). Nrf2 and Keap1 abnormalities in non-small cell lung carcinoma and association with clinicopathologic features. Clin. Cancer Res. *16*, 3743–3753. https://doi.org/10.1158/1078-0432.CCR-09-3352.
31. Yang, H., Wang, W., Zhang, Y., Zhao, J., Lin, E., Gao, J., and He, J. (2011). The Role of NF-E2-Related Factor 2 in Predicting Chemoresistance and Prognosis in Advanced Non–Small-Cell Lung Cancer. Clin. Lung Cancer *12*, 166–171. https://doi.org/10.1016/j.cllc.2011.03.012.
32. Banas, K., Modarai, S., Rivera-Torres, N., Yoo, B.C., Bialk, P.A., Barrett, C., Batish, M., and Kmiec, E.B. (2022). Exon skipping induced by CRISPR-directed gene

editing regulates the response to chemotherapy in non-small cell lung carcinoma cells. Gene Ther. *29*, 357–367. https://doi.org/10.1038/S41434-022-00324-7.

33. Kerins, M.J., and Ooi, A. (2018). A catalogue of somatic NRF2 gain-of-function mutations in cancer. Sci. Rep. *8*, 12846. https://doi.org/10.1038/s41598-018-31281-0.

34. Banas, K., Rivera-Torres, N., Bialk, P., Yoo, B.C., Kmiec, E.B., and Kmiec, E.B. (2020). Kinetics of nuclear uptake and site-specific DNA cleavage during crispr-directed gene editing in solid tumor cells. Mol. Cancer Res. *18*, 891–902. https://doi.org/10.1158/1541-7786.MCR-19-1208.

35. Jin, X., Lou, X., Qi, H., Zheng, C., Li, B., Siwu, X., Liu, R., Lv, Q., Zhao, A., Ruan, J., and Jiang, M. (2024). NRF2 signaling plays an essential role in cancer progression through the NRF2-GPX2-NOTCH3 axis in head and neck squamous cell carcinoma. Oncogenesis *13*, 35. https://doi.org/10.1038/S41389-024-00536-Z.

36. Bae, S., Park, J., and Kim, J.S. (2014). Cas-OFFinder: a fast and versatile algorithm that searches for potential off-target sites of Cas9 RNA-guided endonucleases. Bioinformatics *30*, 1473–1475. https://doi.org/10.1093/BIOINFORMATICS/BTU048.

37. Rangan, S.R.S. (1972). A new human cell line (FaDu) from a hypopharyngeal carcinoma. Cancer *29*, 117–121. https://doi.org/10.1002/1097-0142(197201)29:1<117::AID-CNCR2820290119>3.0.CO;2-R.

38. Fuereder, T. (2022). Essential News of Current Guidelines: Head and Neck Squamous Cell Carcinoma (Springer). https://doi.org/10.1007/s12254-022-00842-5.

39. Wise-Draper, T.M., Bahig, H., Tonneau, M., Karivedu, V., and Burtness, B. (2022). Current Therapy for Metastatic Head and Neck Cancer: Evidence, Opportunities, and Challenges (American Society of Clinical Oncology Educational Book), pp. 527–540. https://doi.org/10.1200/edbk_350442.

40. Zimta, A.A., Cenariu, D., Irimie, A., Magdo, L., Nabavi, S.M., Atanasov, A.G., and Berindan-Neagoe, I. (2019). The Role of Nrf2 Activity in Cancer Development and Progression. Cancers *11*, 1755. https://doi.org/10.3390/CANCERS11111755.

41. Zuo, L., Zou, X., Ge, J., Hu, S., Fang, Y., Xu, Y., Chen, R., Xu, S., Yu, G., Zhou, X., and Ji, L. (2025). The Nrf2-HMOX1 pathway as a therapeutic target for reversing cisplatin resistance in non-small cell lung cancer via inhibiting ferroptosis. Cell Death Discov. *11*, 287. https://doi.org/10.1038/S41420-025-02564-Z.

42. Dziadosz-Brzezińska, A., Kusiński, S., Piróg, A., Urban-Wójciuk, Z., Padariya, M., Kalathiya, U., Kote, S., and Sznarkowska, A. (2025). Considerations for antibody-based detection of NRF2 in human cells. Redox Biol. *81*, 103549. https://doi.org/10.1016/J.REDOX.2025.103549.

43. Shimada, Y., Imamura, M., Wagata, T., Yamaguchi, N., and Tobe, T. (1992). Characterization of 21 newly established esophageal cancer cell lines. Cancer *69*, 277–284. https://doi.org/10.1002/1097-0142(19920115)69:2<277::AID-CNCR2820690202>3.0.CO;2-C.

44. Shen, L.-Y., Wang, H., Dong, B., Yan, W.-P., Lin, Y., Shi, Q., and Chen, K.-N. (2015). Possible prediction of the response of esophageal squamous cell carcinoma to neoadjuvant chemotherapy based on gene expression profiling. Oncotarget *7*, 4531–4541.

45. Tao, S., Wang, S., Moghaddam, S.J., Ooi, A., Chapman, E., Wong, P.K., and Zhang, D.D. (2014). Oncogenic KRAS confers chemoresistance by upregulating NRF2. Cancer Res. *74*, 7430–7441. https://doi.org/10.1158/0008-5472.CAN-14-1439.

46. Cloer, E.W., Goldfarb, D., Schrank, T.P., Weissman, B.E., and Major, M.B. (2019). Nrf2 activation in cancer: From DNA to protein. Cancer Res. *79*, 889–898. https://doi.org/10.1158/0008-5472.CAN-18-2723.

47. Banas, K.H., and Kmiec, E.B. (2025). CRISPR-directed exon skipping; friend or foe (opportunity or warning sign)? Mol. Ther. Nucleic Acids *36*, 102539. https://doi.org/10.1016/J.OMTN.2025.102539.

48. Bloh, K., Kanchana, R., Bialk, P., Banas, K., Zhang, Z., Yoo, B.C., and Kmiec, E.B. (2021). Deconvolution of Complex DNA Repair (DECODR): Establishing a Novel Deconvolution Algorithm for Comprehensive Analysis of CRISPR-Edited Sanger Sequencing Data. CRISPR J. *4*, 120–131. https://doi.org/10.1089/CRISPR.2020.0022.

49. Barsouk, A., Aluru, J.S., Rawla, P., Saginala, K., Barsouk, A., and Barsouk, A. (2023). Epidemiology, Risk Factors, and Prevention of Head and Neck Squamous Cell Carcinoma. Med. Sci. *11*, 42. https://doi.org/10.3390/medsci11020042.

50. Hsu, P.D., Lander, E.S., and Zhang, F. (2014). Development and Applications of CRISPR-Cas9 for Genome Engineering. Cell *157*, 1262–1278. https://doi.org/10.1016/j.cell.2014.05.010.

51. Zimta, A.A., Cenariu, D., Irimie, A., Magdo, L., Nabavi, S.M., Atanasov, A.G., and Berindan-Neagoe, I. (2019). The role of Nrf2 activity in cancer development and progression. Cancers *11*, 1755. https://doi.org/10.3390/cancers11111755.

52. Rojo de la Vega, M., Chapman, E., and Zhang, D.D. (2018). NRF2 and the Hallmarks of Cancer. Cancer Cell *34*, 21–43.

53. Syu, J.-P., Chi, J.-T., and Kung, H.-N. (2016). Nrf2 Contributes to the Poor Prognosis and Chemoresistance. In A Master Regulator of Oxidative Stress - the Transcription Factor Nrf2 (InTech). https://doi.org/10.5772/65105.

54. Panieri, E., and Saso, L. (2019). Review Article Potential Applications of NRF2 Inhibitors in Cancer Therapy. Oxid. Med. Cell. Longev. *2019*, 8592348. https://doi.org/10.1155/2019/8592348.

55. Hunt, J.M.T., Samson, C.A., du Rand, A. du, and Sheppard, H.M. (2023). Unintended CRISPR-Cas9 editing outcomes: a review of the detection and prevalence of structural variants generated by gene-editing in human cells. Hum. Genet. *142*, 705–720. https://doi.org/10.1007/s00439-023-02561-1.

56. Guo, C., Ma, X., Gao, F., and Guo, Y. (2023). Off-target effects in CRISPR/Cas9 gene editing. Front. Bioeng. Biotechnol. *11*, 1143157. https://doi.org/10.3389/fbioe.2023.1143157.

57. Liu, M., Zhang, W., Xin, C., Yin, J., Shang, Y., Ai, C., Li, J., Meng, F.-L., and Hu, J. (2021). Global detection of DNA repair outcomes induced by CRISPR-Cas9. Nucleic Acids Res. *49*, 8732–8742. https://doi.org/10.1101/2021.02.15.431335.

OMTON, Volume *34*

Supplemental information

Target choice and exon skipping regulate CRISPR-directed gene editing of NRF2 in head/neck and esophageal cancer cells

Natalia Rivera-Torres, Lauren E. Skelly, John A. Rogowskyj, Guadalupe Aguilar, Kelly Banas, Pawel Bialk, and Eric B. Kmiec

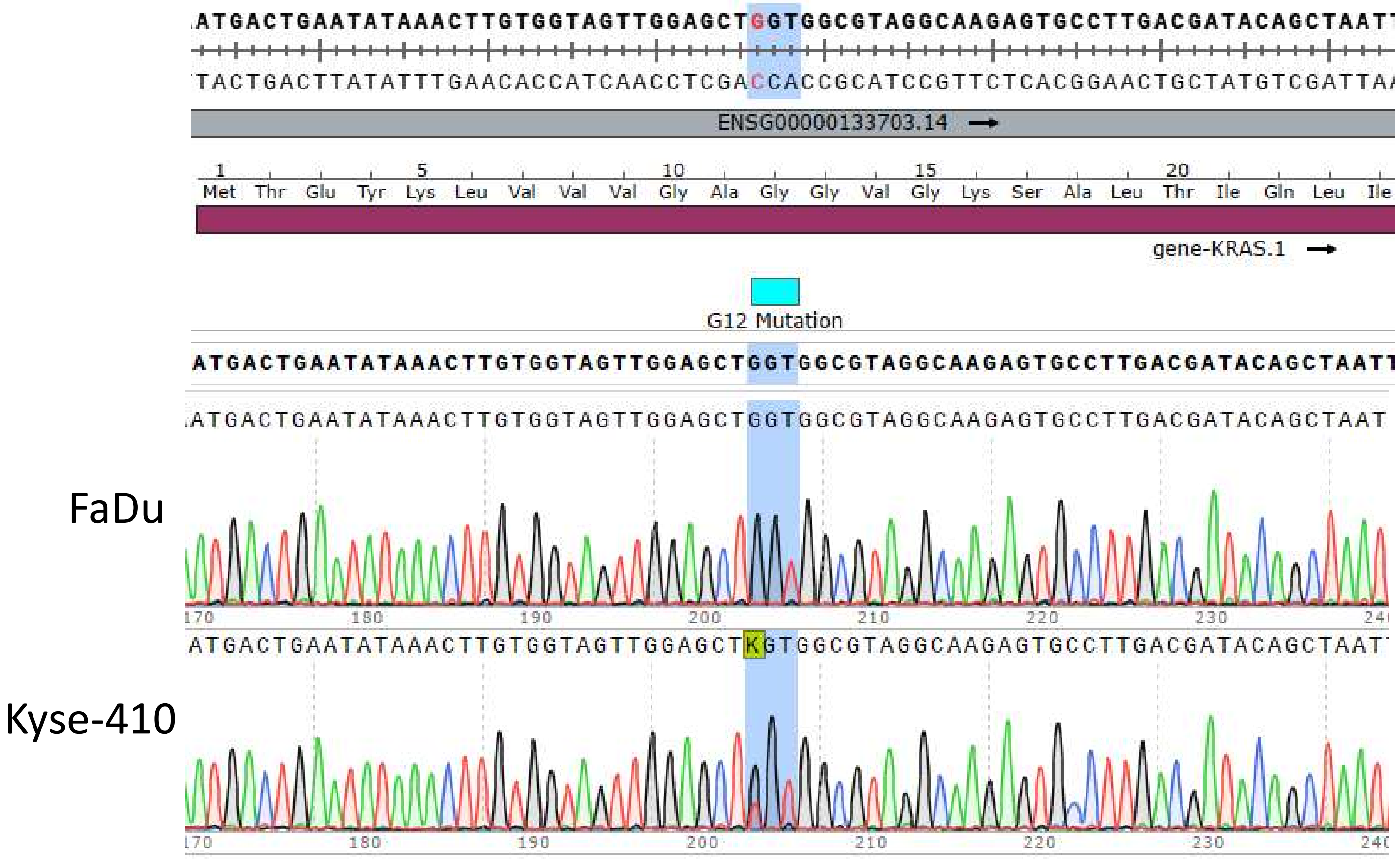

Figure S1. **KRAS Genotyping**. Sanger sequencing results of the KRAS gene in FaDu and KYSE410 cells. The G12D mutation was detected in the KYSE410 cells.

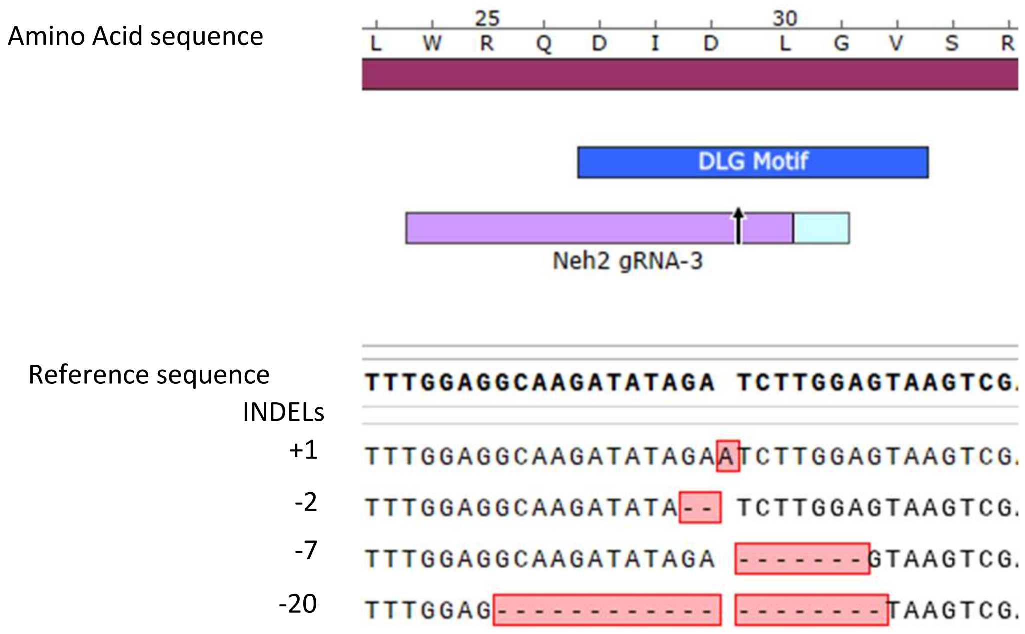

Figure S2. NRF2 interacts with KEAP-1 via the DLG amino acid motif encoded by exon 2. Major INDELS resulting from sgRNA3 targeting directly disrupts sequence.

FaDu

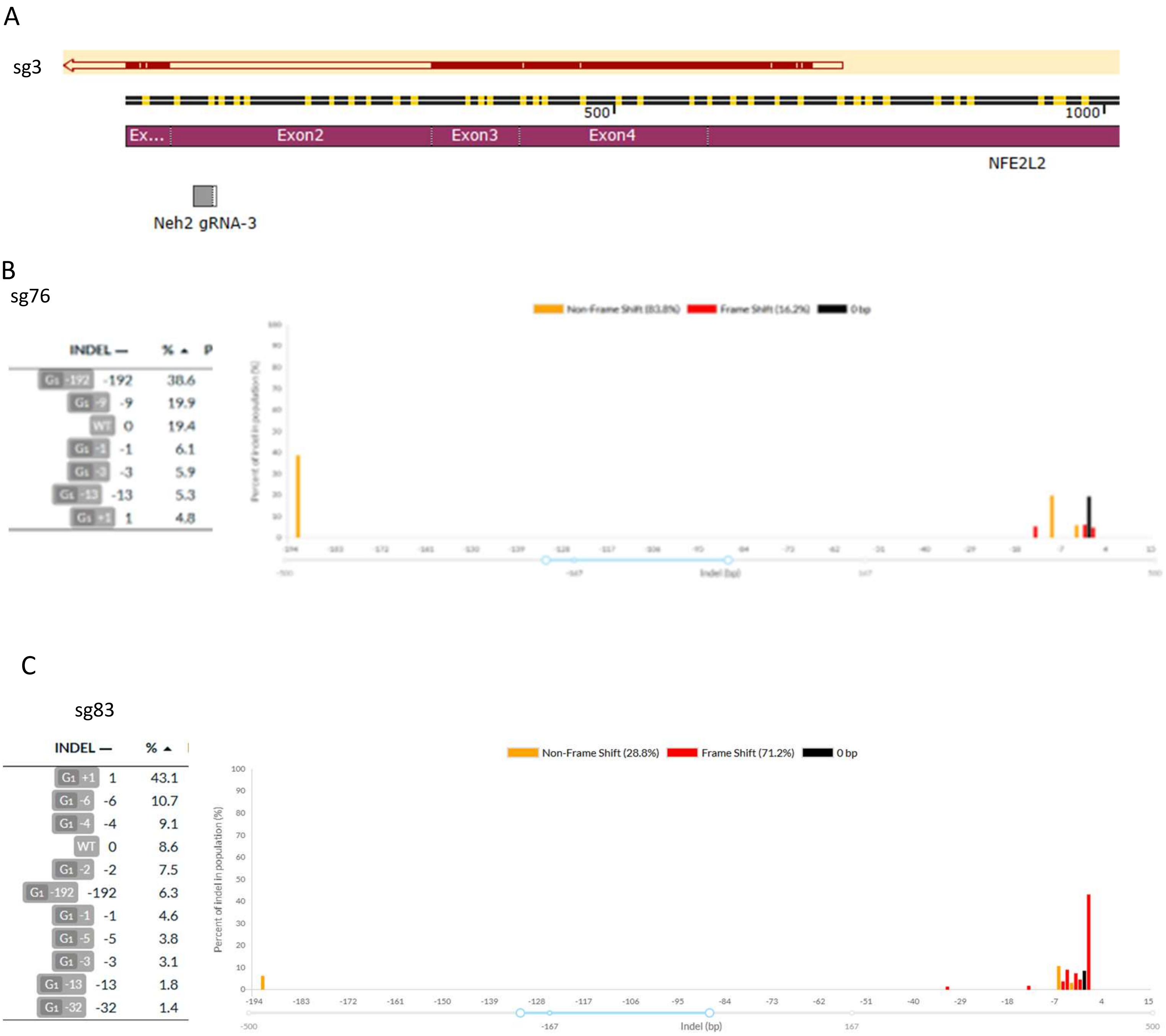

Figure S3. **cDNA sequencing to assess Exon skipping in FADU cells (A)** cDNA from FaDu cells targeted with sg3 in NRF2 was sanger sequenced and aligned to the reference cDNA sequence. cDNA from FaDu cells targeted with sg76 **(B)** or sg83 **(C)** was sanger sequenced and visualized in Decodr (www.decodr.org)

KYSE

A

sg3

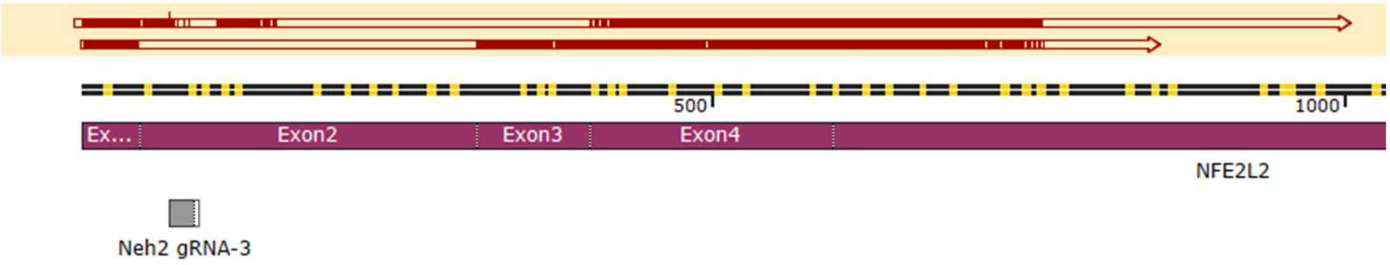

B

sg76

INDEL —		% ▲
G1 -1	-1	41.7
G1 -193	-193	31.2
G1 -10	-10	14.3
G1 -277	-277	7.6
G1 -40	-40	5.1

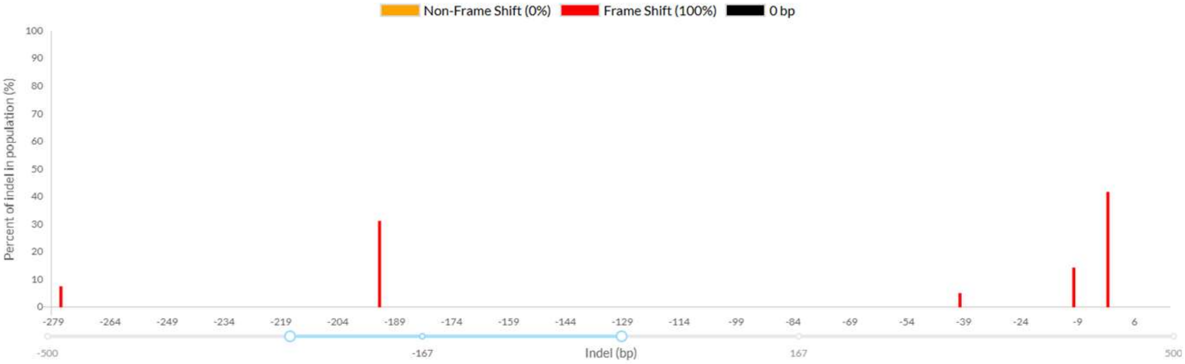

sg83

C

INDEL —		% ▲
G1 +1	1	24.0
G1 -192	-192	19.9
G1 -6	-6	18.6
WT	0	18.4
G1 -4	-4	7.5
G1 -14	-14	6.9
G1 -2	-2	4.7

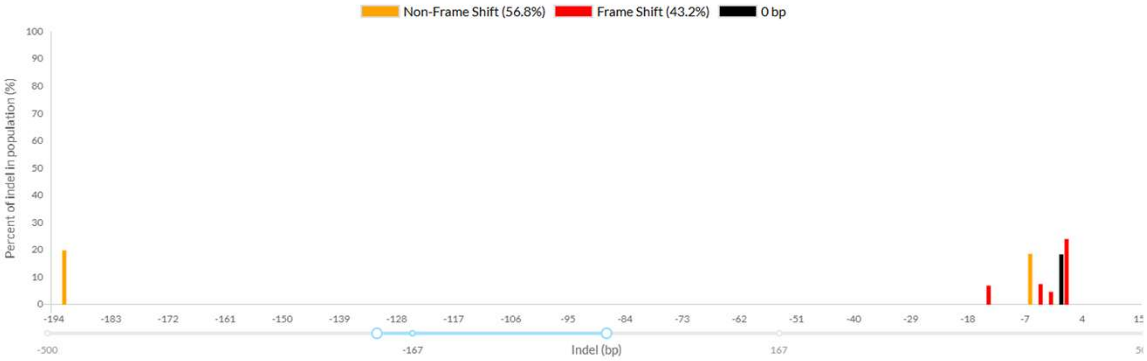

Figure S4.cDNA sequencing to assess Exon skipping in KYSE410 cells.(A) cDNA from KYSE cells targeted with sg3 in NRF2 was sanger sequenced and aligned to the reference cDNA sequence. cDNA from KYSE cells targeted with sg76 **(B)** or sg83 **(C)** was sanger sequenced and visualized in Decodr (www.decodr.org)

Aligned Sequence	INDEL	72hr%	2wk%	%Frameshift 72hr	% Frameshift 2wk
**sg3** TGGAGGCAAGATATAGATCTTGG	Total	85.51	73.45	78.2	74.9
GATTGACATACTTTGGAGGCAAGATATAGATCTTGGAGTAAGTCGAGAAGTATTTGACTT	0	14.49	26.55		
GATTGACATACTTTGGAG--------------------TAAGTCGAGAAGTATTTGACTT	-20	6.07	4.70		
GATTGACATACTTTGGAGGCAAGATATAG**A**ATCTTGGAGTAAGTCGAGAAGTATTTGACT	1	5.70	3.95		
GATTGACATACTTTGGAGGCAAGATATAGA-------GTAAGTCGAGAAGTATTTGACTT	-7	5.30	4.04		
GATTGACATACTTTGGAGGCAAGATATA--TCTTGGAGTAAGTCGAGAAGTATTTGACTT	-2	4.00	4.26		
GATTGACATACTTTGGAGGCAAGATATAGA-CTTGGAGTAAGTCGAGAAGTATTTGACTT	-1	2.87	2.04		
GATTGACATACTTTGGAGGCAAGATAT----CTTGGAGTAAGTCGAGAAGTATTTGACTT	-4	2.67	1.99		
GATTGACATACTTTGGAGGCAAGATATAG-TCTTGGAGTAAGTCGAGAAGTATTTGACTT	-1	2.39	1.72		
GATTGACATACTTTGGAGGCAAG-------------------TCGAGAAGTATTTGACTT	-19	2.25	1.68		
GATTGACATACTTTGGAGGCAAGATATAGA--TTGGAGTAAGTCGAGAAGTATTTGACTT	-2	1.99	1.84		
GATTGACATACTTTGGAGGCAAGATATAGA--------TAAGTCGAGAAGTATTTGACTT	-8	1.84	1.05		
GATTGACATACTTTGGAGGCAAGATATAGA**T**TCTTGGAGTAAGTCGAGAAGTATTTGACT	1	1.77	2.11		
GATTGACATACTTTGGAGGCAAGAT------CTTGGAGTAAGTCGAGAAGTATTTGACTT	-6	1.57	1.29		
GATTGACATACTTTGGAGGCAAGAT-----TCTTGGAGTAAGTCGAGAAGTATTTGACTT	-5	1.37	0.77		
GATTGACATACTTTGGAGGCAAGATAT---TCTTGGAGTAAGTCGAGAAGTATTTGACTT	-3	1.16	1.11		
other		44.57	40.90		
**sg76** GTCACTTGTTCCTGATATTCCCGG	Total	87.94	49.30	68.9	66.6
GGCTACGTTTCAGTCACTTGTTCCTGATAT**T**TCCCGGTCACATCGAGAGC	1	13.10	6.13		
GGCTACGTTTCAGTCACTTGTTCCTGATATTCCCGGTCACATCGAGAGCC	0	12.06	50.70		
GGCTACGTTTCAGTCACTTGTTCCTGATAT-CCCGGTCACATCGAGAGCC	-1	10.97	7.19		
GGCTACGTTTCAGTCACTTGTTCC---------CGGTCACATCGAGAGCC	-9	8.87	5.09		
GGCTACGTTTCAGTCACTTGTTCCTGAT--TCCCGGTCACATCGAGAGCC	-2	3.17	1.81		
GGCTACGTTTCAGTCACTTGT----------CCCGGTCACATCGAGAGCC	-10	2.08	1.06		
GGCTACGTTTCAGTCACTTGTTCCTGAT---------CACATCGAGAGCC	-9	2.00	0.80		
GGCTACGTTTCAGTCACTTGTTCCTGATAT------------CGAGAGCC	-12	1.77	1.21		
GGCTACGTTTCAGTCACTTGTTCC----------GGTCACATCGAGAGCC	-10	1.47	0.34		
GGCTACGTTTCAGTCACTTGTTCCTG---------GTCACATCGAGAGCC	-9	1.39	0.46		
GGCTACGTTTCAGTCACTTGTTCCTGAT---CCCGGTCACATCGAGAGCC	-3	1.39	0.88		
GGCTACGTTTCAGTCACTTGTTCCTG----TCCCGGTCACATCGAGAGCC	-4	1.30	0.74		
GGCTACGTTTCAGTCAC-----------------------ATCGAGAGCC	-23	1.26	0.24		
GGCTACGTTTCAGTCACTTGTTCCTGATAT**AT**TCCCGGTCACATCGAGAG	2	1.09	0.70		
other		38.08	22.66		
**sg83** GTAGCCCCTGTTGATTTAGACGG	Total	86.20	40.95	70.9	67.8
TTCTGTTGCTCAGGTAGCCCCTGTTGATTT**T**AGACGGTATGCAACAGGAC	1	18.19	8.61		
TTCTGTTGCTCAGGTAGCCCCTGTTGATTTAGACGGTATGCAACAGGACA	0	13.80	59.05		
TTCTGTTGCTCAGGTAGCCCCTGTTGA------CGGTATGCAACAGGACA	-6	8.53	4.01		
TTCTGTTGCTCAGGTAGCCCCTGTTGA----GACGGTATGCAACAGGACA	-4	3.81	1.60		
TTCTGTTGCTCAGGTAGCCCCTGTTGATTT**TT**AGACGGTATGCAACAGGA	2	3.20	0.57		
TTCTGTTGCTCAGGTAGCCCCTGTTGATT-AGACGGTATGCAACAGGACA	-1	2.40	1.44		
TTCTGTTGCTCAGGTAGCCCCTGTTGAT--AGACGGTATGCAACAGGACA	-2	2.35	0.83		
TTCTGTTGCTCAGGTAGCCCCTGTTG---TAGACGGTATGCAACAGGACA	-3	1.84	1.00		
TTCTGTTGCTCAGGTAGCCCCTGT-------------ATGCAACAGGACA	-13	1.64	0.37		
TTCTGTTGCTCAGGTAGCCCCTGTTGATTT----------CAACAGGACA	-10	1.43	0.35		
TTCTGTTGCTCAGGTAGCCCCTGTTGA---AGACGGTATGCAACAGGACA	-3	1.25	0.28		
TTCTGTTGCTCAGGTAG---------------ACGGTATGCAACAGGACA	-15	1.21	0.52		
TTCTGTTGCTCAG-------------------ACGGTATGCAACAGGACA	-19	1.19	0.43		
TTCTGTTGCTCAGGTAGCCCCTGTTGATTT-GACGGTATGCAACAGGACA	-1	1.16	0.32		
TTCTGTTGCTCAGGTA----------------------TGCAACAGGACA	-22	1.03	0.69		
other		36.98	19.94		

Figure S5. **Genomic analyses of NRF2 2 weeks post CRISPR/Cas9 targeting.** Genomic DNA from FaDu cells was isolated and amplified across exon 2 and exon 4 of the *NRF2* gene 13 days after transfection. Amplicon was NGS sequenced and analyzed for indels at the CRISPR target site. Raw sequence files were aligned using the software program, CRISPResso2, to display the *NRF2* allele-specific indel pattern

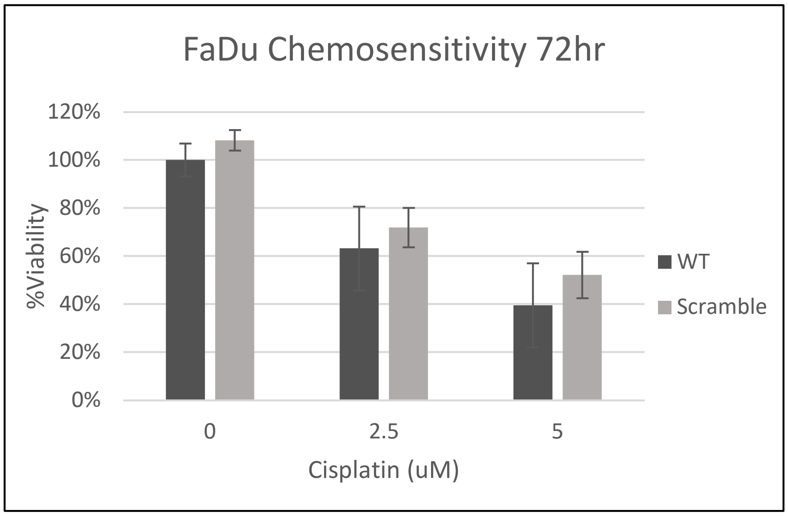

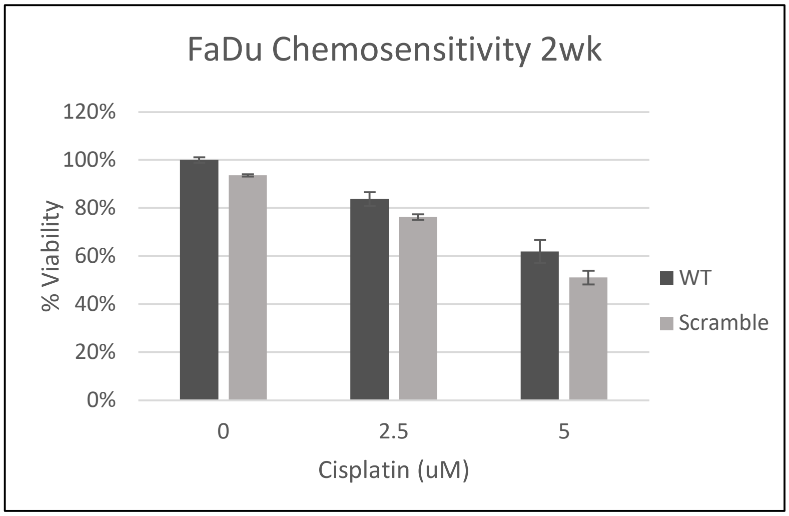

Figure S6. **Sustained Chemosensitivity Testing 2 weeks after CRISPR/Cas9 targeting of NRF2.** Chemosensitivity was measured via CellTiter-Glo® 2.0 Assay. Targeted cells that were maintained in culture for 2 weeks post-transfection was treated with increasing concentrations of cisplatin for 72 hr. and then evaluated for cell viability. The average relative viability of cells normalized to the untreated Wt was graphed. The error bars represent the Coefficient of variance.
